# High 4E-BP1 expression associates with chromosome 8 gain and CDK4/6 sensitivity in Ewing sarcoma

**DOI:** 10.1172/JCI187627

**Published:** 2025-10-16

**Authors:** Cornelius M. Funk, Anna C. Ehlers, Martin F. Orth, Karim Aljakouch, Jing Li, Tilman L.B. Hölting, Rainer Will, Florian H. Geyer, A. Katharina Ceranski, Franziska Willis, Endrit Vinca, Shunya Ohmura, Roland Imle, Jana Siebenlist, Angelina Yershova, Maximilian M.L. Knott, Felina Zahnow, Ana Sastre, Javier Alonso, Felix Sahm, Heike Peterziel, Anna Loboda, Martin Schneider, Ana Banito, Gabriel Leprivier, Wolfgang Hartmann, Uta Dirksen, Olaf Witt, Ina Oehme, Stefan M. Pfister, Laura Romero-Pérez, Jeroen Krijgsveld, Florencia Cidre-Aranaz, Thomas G.P. Grünewald, Julian Musa

**Affiliations:** 1 German Cancer Research Center (DKFZ) Heidelberg, Division of Translational Pediatric Sarcoma Research, Heidelberg, Germany.; 2 Hopp Children’s Cancer Center Heidelberg (KiTZ), Heidelberg, Germany.; 3 National Center for Tumor Diseases (NCT) Heidelberg, a partnership between DKFZ and Heidelberg University Hospital, Heidelberg, Germany.; 4 Max-Eder Research Group for Pediatric Sarcoma Biology, Institute of Pathology, Faculty of Medicine, Ludwig Maximilian University (LMU) Munich, Munich, Germany.; 5 DKFZ Heidelberg, Division of Proteomics of Stem Cells and Cancer, Heidelberg, Germany.; 6 Medical Faculty, Heidelberg University, Heidelberg, Germany.; 7 Department of Medical Oncology, NCT, Heidelberg, Germany.; 8 Cellular Tools Core Facility, DKFZ, German Cancer Consortium (DKTK), Heidelberg, Germany.; 9 Department of General, Visceral, and Transplant Surgery, University Hospital Heidelberg, Heidelberg, Germany.; 10 Department of General, Visceral, Thoracic, and Transplant Surgery, University Hospital Giessen and Marburg, Giessen, Germany.; 11 Soft-Tissue Sarcoma Junior Research Group, DKFZ, Heidelberg, Germany.; 12 Division of Pediatric Surgery, University Hospital Heidelberg, Heidelberg, Germany.; 13 Faculty of Biosciences, Heidelberg University, Heidelberg, Germany.; 14 Institute of Pathology, Faculty of Medicine, LMU Munich, Munich, Germany.; 15 Unidad Hemato-oncología Pediátrica, Hospital Infantil Universitario La Paz, Madrid, Spain.; 16 Pediatric Solid Tumour Laboratory, Institute of Rare Diseases Research (IIER) and; 17 Centro de Investigación Biomédica en Red de Enfermedades Raras, Instituto de Salud Carlos III (CB06/07/1009; CIBERER-ISCIII), Madrid, Spain.; 18 DKFZ Heidelberg, Clinical Cooperation Unit Neuropathology, Heidelberg, Germany.; 19 Department of Neuropathology, University Hospital Heidelberg, Heidelberg, Germany.; 20 Department of Pediatric Oncology, Hematology, Immunology and Pulmonology, Heidelberg University Hospital, Heidelberg, Germany.; 21 Faculty of Biosciences, Heidelberg University, Heidelberg, Germany.; 22 Institute of Neuropathology, University Hospital Düsseldorf, Medical Faculty, Heinrich Heine University, Düsseldorf, Germany.; 23 Division of Translational Pathology, Gerhard-Domagk-Institute of Pathology, University Hospital of Münster, Münster, Germany.; 24 Pediatrics III, West German Cancer Center, German Cancer Consortium site Essen, NCT site Essen, University Hospital of Essen, Essen, Germany.; 25 DKFZ Heidelberg, Clinical Cooperation Unit Pediatric Oncology, Germany.; 26 DKFZ Heidelberg, Division of Pediatric Neurooncology, Germany.; 27 Division of Molecular Pathology of Sarcomas, Institute of Biomedicine of Sevilla (IBiS), Virgen del Rocio University Hospital/CSIC/University of Sevilla/CIBERONC, Seville, Spain.; 28 Department of Normal and Pathological Cytology and Histology, School of Medicine, University of Seville, Seville, Spain.; 29 Institute of Pathology, Heidelberg University Hospital, Heidelberg, Germany.

**Keywords:** Genetics, Oncology, Cancer, Genetic variation, Mouse models

## Abstract

Chromosome 8 (chr8) gains are common in cancer, but their contribution to tumor heterogeneity is largely unexplored. Ewing sarcoma (EwS) is defined by *FET::ETS* fusions with few other recurrent mutations to explain clinical diversity. In EwS, chr8 gains are the second most frequent alteration, making it an ideal model to study the relevance of chr8 gains in an otherwise silent genomic context. We report that chr8 gain–driven expression patterns correlate with poor overall survival of patients with EwS. This effect is mainly mediated by increased expression of the translation initiation factor binding protein 4E-BP1, encoded by *EIF4EBP1* on chr8. Among all chr8-encoded genes, *EIF4EBP1* expression showed the strongest association with poor survival and correlated with chr8 gains in EwS tumors. Similar findings emerged across multiple cancer entities in The Cancer Genome Atlas. Multiomics profiling revealed that 4E-BP1 orchestrates a pro-proliferative proteomic network. Silencing 4E-BP1 reduced proliferation, clonogenicity, spheroidal growth in vitro, and tumor growth in vivo. Drug screens demonstrated that high 4E-BP1 expression sensitizes EwS to pharmacological CDK4/6-inhibition. Chr8 gains and elevated 4E-BP1 emerge as prognostic biomarkers in EwS, with poor outcomes driven by 4E-BP1–mediated pro-proliferative networks that sensitize tumors to CDK4/6 inhibitors. Testing for chr8 gains may enhance risk stratification and therapy in EwS and other cancers.

## Abstract 2

We show how recurrent chromosome 8 gains drive poor patient outcomes via 4E-BP1 sensitizing for CDK4/6-inhibition in the Ewing sarcoma model.

## Introduction

Aneuploidy is common in cancer cells and plays an important functional role in their pathophysiology (1–3). Copy number alterations of chromosome 8 (chr8), especially chr8 gains, are observed in numerous cancer entities, including Ewing sarcoma (EwS), acute and chronic myeloid leukemia, gastric cancer, myxoid liposarcoma, pediatric undifferentiated sarcoma, clear cell sarcoma, and malignant peripheral nerve sheath tumors (2, 4–11). However, the functional and clinical role of chr8 gains remains to be clarified. In the context of precision oncology, understanding the role of specific chromosomal gains and losses as a major source of inter-tumor heterogeneity is important for the development of novel personalized diagnostic and therapeutic approaches.

EwS is a malignant bone- and soft tissue–associated tumor, primarily occurring in children, adolescents, and young adults (12). It is characterized by a low number of recurrent somatic mutations and is driven by chromosomal translocations generating pathognomonic *FET::ETS* fusions (consisting of members from the *FUS*/*EWS*/*TAF15* [FET] gene family and the E26 transformation-specific [ETS] gene family) with Ewing sarcoma breakpoint region 1:Friend leukemia integration 1 (*EWSR1::FLI1*) being the most common (present in 85% of cases), encoding aberrant chimeric transcription factors (12). Genetic variants in polymorphic enhancer-like DNA binding sites of EWSR1::FLI1 account for inter-individual heterogeneity in EwS susceptibility, tumor growth, clinical course, and treatment response (13–15). Secondary somatic mutations in *STAG2* and *TP53* occur in approximately 20% and 5% of patients with EwS, respectively (16–18). However, little is known about other, even more common recurrent alterations, such as chromosomal gains and/or losses and their impact on interindividual tumor heterogeneity.

Chr8 gain is present in approximately 50% of EwS cases, often in the form of chr8 trisomy, making it the second most frequently observed recurrent somatic alteration in EwS, after *FET::ETS* fusions (16–22). Previous studies focused solely on specific correlations regarding the role of (partial) chr8 gains in EwS (16–19, 21, 23–28) and suggested that chr8 gains may be an early event in EwS tumorigenesis (29). However, the precise functional and clinical impact of whole chr8 gains in EwS remains unclear. EwS is an ideal model to investigate the role of chr8 gain in cancer, given that EwS exhibits a “silent” genome in which chr8 gains occur in an oligomutated genomic context (12).

Therefore, in the present study, we investigated the possible association between whole chr8 gains and tumor progression in the EwS model and aimed to identify the most clinically relevant genes located on chr8 that may functionally contribute to interindividual variability in patient outcomes. Following an integrative functional genomics approach, we identified the eukaryotic translation initiation factor 4E binding protein 1 (EIF4EBP1, alias 4E-BP1) as the most promising chr8 candidate gene. It is outstandingly associated with unfavorable outcomes for patients with EwS compared with all other captured genes located on chr8 and even across the entire EwS transcriptome. 4E-BP1 functions downstream of its inactivating kinase complex, mTORC complex 1 (mTORC1), and is a key effector of the mTORC1 signaling pathway (30, 31). 4E-BP1 belongs to a family of eIF4E-binding proteins that enable mTORC1 to adjust mRNA translation rates in response to various stimuli by modulating the assembly of the 48S translation initiation complex (30, 32–34). 4E-BP1 essentially blocks overall cap-dependent mRNA translation rates but also exerts selectivity in promoting and inhibiting translation of specific transcripts (30, 34–42). However, its precise and maybe dynamic role in tumor initiation and/or progression is still ambiguous and a current matter of debate since tumor-suppressing (43–45) and tumor-promoting roles of 4E-BP1 have been described depending on the cancer entity and cellular context (30, 37, 42, 45, 46).

In the present study, we demonstrate that overexpression of *EIF4EBP1* is mediated by chr8 gain in primary EwS tumors. Furthermore, its RNAi-mediated knockdown in cell line models reduces EwS growth in vitro and in vivo by influencing a pro-proliferative proteomic network. Thus, we establish an association between chr8 gain and tumor progression, mediated by 4E-BP1 in EwS. Drug screens and drug sensitivity assays in vitro and in vivo revealed that high 4E-BP1 expression sensitizes cells to targeted CDK4/6 inhibitor treatment with the FDA-approved drugs palbociclib and ribociclib. This discovery offers a therapeutic strategy for tumors with chr8 amplification and 4E-BP1 overexpression.

## Results

### Chr8 gain drives overexpression of the clinically relevant translational regulator 4E-BP1 in EwS.

**Figure 1:**
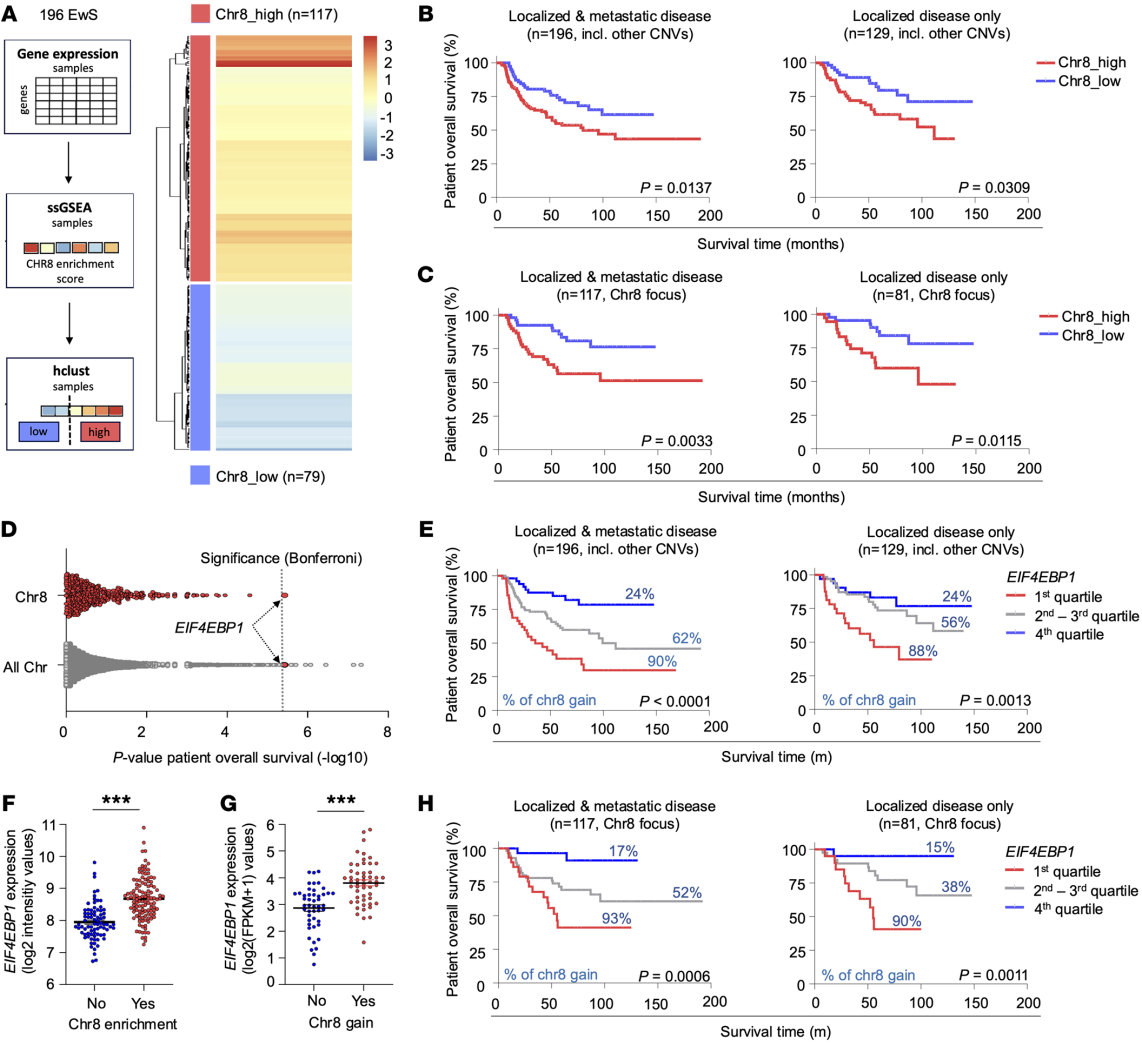
Chr8 gain drives overexpression of the clinically relevant translation initiation factor 4E-BP1 in EwS. (**A**) Flowchart illustrating patient stratification to either a high or low chr8 gene-expression-signature group based on hierarchical clustering (hclust) of sample-specific ssGSEA enrichment scores for the chr8 gene set (left). Heatmap of ssGSEA scores for chr8 genes in 196 EwS tumors (cohort 1) (right). Color intensity indicates the degree of gene set enrichment. (**B**) Kaplan-Meier overall survival analysis of cohort 1 stratified into either a high or low chr8-signature-enrichment group as described in (**A**). *P* values were determined by the Mantel-Haenszel test. (**C**) Kaplan-Meier overall survival analysis of 117 patients with EwS (cohort 1 with Chr8 focus) stratified into either a high or low chr8-signature-enrichment group as described in (**A**) but excluding samples with other inferred, recurrent CNVs. *P* values were determined by the Mantel-Haenszel test. (**D**) Overall survival batch analysis as assessed for every gene or exclusively chr8 genes covered in transcriptomic profiling of cohort 1 using the Mantel-Haenszel test. (**E**) Kaplan-Meier overall survival analysis of cohort 1 stratified by quartile of *EIF4EBP1* expression. Percentages given for each expression quartile refer to the percentage of patients with predicted chr8 gain in the respective quartile. *P* values were determined by the Mantel-Haenszel test. (**F**) *EIF4EBP1* expression as measured by microarray profiling in 196 EwS tumors (cohort 1) stratified into either a high or low chr8-signature-enrichment group as described in (**A**). *P* values were determined by 2-tailed Mann-Whitney test; horizontal bars represent means, and whiskers represent the SEM. ***P < 0.001. (**G**) *EIF4EBP1* expression as measured by RNA-Seq in cohort 2 depending on the presence of chr8 gain as determined by methylation array. *P* values were determined by 2-tailed Mann-Whitney test; horizontal bars represent means, and whiskers represent the SEM. ***P < 0.001. (**H**) Kaplan-Meier overall survival analysis of 117 patients with EwS (cohort 1 with Chr8 focus, as in **C**) stratified by quartile of *EIF4EBP1* expression. Percentages given for each expression quartile refer to the percentage of patients with predicted chr8 gain in the respective quartile. *P* values were determined by the Mantel-Haenszel test.

To gain initial insights into whether chr8 gain mediates poor patient outcomes in EwS, we analyzed 196 EwS tumor samples for which matched microarray gene expression data and clinical data were available (cohort 1). We used a chr8 gene expression signature as a surrogate model for factual genomic chr8 gain and performed a single-sample Gene Set Enrichment Analysis (ssGSEA) followed by hierarchical clustering (hclust) (Figure 1A) (47, 48). Each patient was stratified to either a high or low chr8 gene expression signature group based on hierarchical clustering of sample-specific ssGSEA enrichment scores for the chr8 gene set (Figure 1A). To validate our approach, we first identified the differentially expressed genes (DEGs) between the inferred chr8-high and -low clusters. We then performed a position-related data analysis, which identifies chromosomal location of the respective DEGs and maps them to the respective chromosomal positions (49, 50). The position-related data analysis demonstrated that the vast majority of DEGs map to chr8, validating the inferred chr8 signature (Supplemental Figure 1A; supplemental material available online with this article; https://doi.org/10.1172/JCI187627DS1).

Second, we applied our approach to RNA-Seq data from an independent cohort of 100 EwS tumors (cohort 2) and compared the chr8 signature enrichment clustering with matched factual chr8 copy number variation (CNV) status (inferred from DNA methylation arrays). This analysis showed that clustering based on the chr8 gene expression signature enrichment accurately indicates the presence of chr8 gain (Supplemental Figure 1B).

Kaplan-Meier analysis of data from cohort 1 revealed that a high chr8 gene expression signature was associated with shorter overall survival among patients with EwS (*P* = 0.0137) (Figure 1B). Strikingly, this association remained significant (*P* = 0.0309) (Figure 1B) even when only considering patients with localized disease (i.e., without evidence for metastasis at diagnosis), indicating that chr8 gain is functionally involved in mediating an unfavorable disease phenotype. In support of this hypothesis, it is intriguing that while chr8 gain is only found in approximately 50% of primary tumors, approximately 80% of EwS cell lines, which are expected to be derived from highly aggressive tumor clones, exhibit chr8 gains (mostly trisomies) (16–19, 21, 24–28). Because previous studies have reported that chr8 gains can co-occur with other recurrent chromosomal gains and losses that may have an effect on patient overall survival (16–18, 27), such as chr1q gains, chr12 gains, and 16q loss, we reanalyzed our cohort 1 to focus on those patients with an exclusive predicted chr8 gain versus those harboring none of the aforementioned CNVs, to rule out possible confounding other gains and losses. As shown in Figure 1C, this yielded even a better patient-stratification regarding overall survival in both localized disease and the entire sub-cohort (*P* = 0.0115 and *P* = 0.0033, respectively). Together, these findings suggest that genes located on chr8 contribute to aggressive cellular behavior and disease progression in EwS.

Previous reports suggested that *MYC* located on chr8 may mediate the effect of chr8 gains on patient outcome in EwS and other undifferentiated sarcomas (10, 51). However, in our large EwS cohort 1, *MYC* expression was not significantly associated with overall patient survival (*P* = 0.689) (Supplemental Figure 1C and Supplemental Table 1). Similarly, the chr8-located gene *RAD21*, previously reported to promote EwS tumorigenicity by mitigating EWSR1::FLI1–induced replication stress (23), was not significantly associated with overall survival of patients with EwS (*P* = 0.174) (Supplemental Figure 1C and Supplemental Table 1). These findings suggest the mechanisms underlying the association of chr8 gain with EwS aggressiveness are more complex than previously anticipated.

To identify chr8-encoded genes most strongly associated with poor overall survival among patients with EwS, we conducted a batch analysis within cohort 1. Using our custom code software, GenEx, we calculated *P* values for the association between gene expression and overall survival for all microarray-represented genes, using the Mantel-Haenszel test (Supplemental Table 1). Among all chr8-located genes analyzed, *EIF4EBP1* expression showed the strongest association with patient outcome, with high *EIF4EBP1* expression significantly correlating with unfavorable overall survival (nominal *P* < 0.0001; Bonferroni-adjusted *P* = 0.049) (Figure 1, D and E, and Supplemental Table 2). High *EIF4EBP1* expression remained significantly associated with poor overall survival even when considering only patients with localized disease (*P* = 0.0013) (Figure 1E). Additionally, *EIF4EBP1* ranked within the top 15 survival-associated genes genome-wide (Figure 1D and Supplemental Table 1). These results are consistent with the association of chr8 gain with poor overall survival among patients with EwS (Figure 1, B and C) as well as with previous research that has linked chr8p, where *EIF4EBP1* is located, with EwS relapse (51, 52). Furthermore, *EIF4EBP1* expression was significantly correlated with high ssGSEA enrichment scores for chr8 gene expression in cohort 1 (*P* < 0.001; *r* = 0.47, by Pearson’s test; Cohen′s *d* = 1.19) (Supplemental Table 3). This suggests that a significant part of the negative prognostic effect of the high chr8 gene expression signature can be attributed to high *EIF4EBP1* expression. Accordingly, the predicted chr8 gain was significantly associated with elevated *EIF4EBP1* expression levels in this cohort (*P* < 0.001) (Figure 1F), which also holds true when only considering patients with an exclusive predicted chr8 gain or none of the other aforementioned CNVs (*P* < 0.001) (Supplemental Figure 1D). This association was confirmed on mRNA and protein levels in the independent cohort 2, with chr8 status detected at the DNA level (*P* < 0.001) (Figure 1G and Supplemental Figure 1E).

Similar to our analyses shown in Figure 1C, we reanalyzed our survival data from cohort 1, now only focusing on exclusively predicted chr8-gained samples versus samples without any recurrent chromosomal gain or loss. The reanalysis fully confirmed the prognostic role of *EIF4EBP1* in patients with EwS (Figure 1H). Interestingly, DEG analysis of cohort 1 comparing chr8-high and -low gene expression revealed that among genes of the mTOR signaling pathway, *EIF4EBP1* is distinctively upregulated in tumors with chr8 gain (Supplemental Figure 1F), indicating that 4E-BP1 has a distinct clinical and functional role within the mTOR signaling pathway in EwS.

To evaluate the potential clinical and functional significance of chr8 gain and *EIF4EBP1* expression in other cancer entities besides EwS, we analyzed CNV data from DNA methylation arrays of The Cancer Genome Atlas (TCGA). Our analysis revealed that numerous cancer entities exhibit chr8 gains (specifically, 8 of 32 identified entities exhibited chr8 gains in >10% of cases) (Supplemental Table 4). Additionally, consistent with previously published data (46), chr8 gain and high *EIF4EBP1* expression are associated with unfavorable patient survival in several other cancer entities (chr8 gain in 4 and high *EIF4EBP1* expression in 14 of 32 identified entities) (Supplemental Table 4). These include hepatocellular carcinoma, renal papillary cell carcinoma, lower-grade glioma, and thymoma (Supplemental Table 4).

Collectively, these results indicate that chr8 gain contributes to unfavorable outcomes in patients with EwS and identify 4E-BP1, encoded on chr8, as a potential driver of EwS aggressiveness.

### 4E-BP1 drives a proliferation-associated proteomic network.

**Figure 2:**
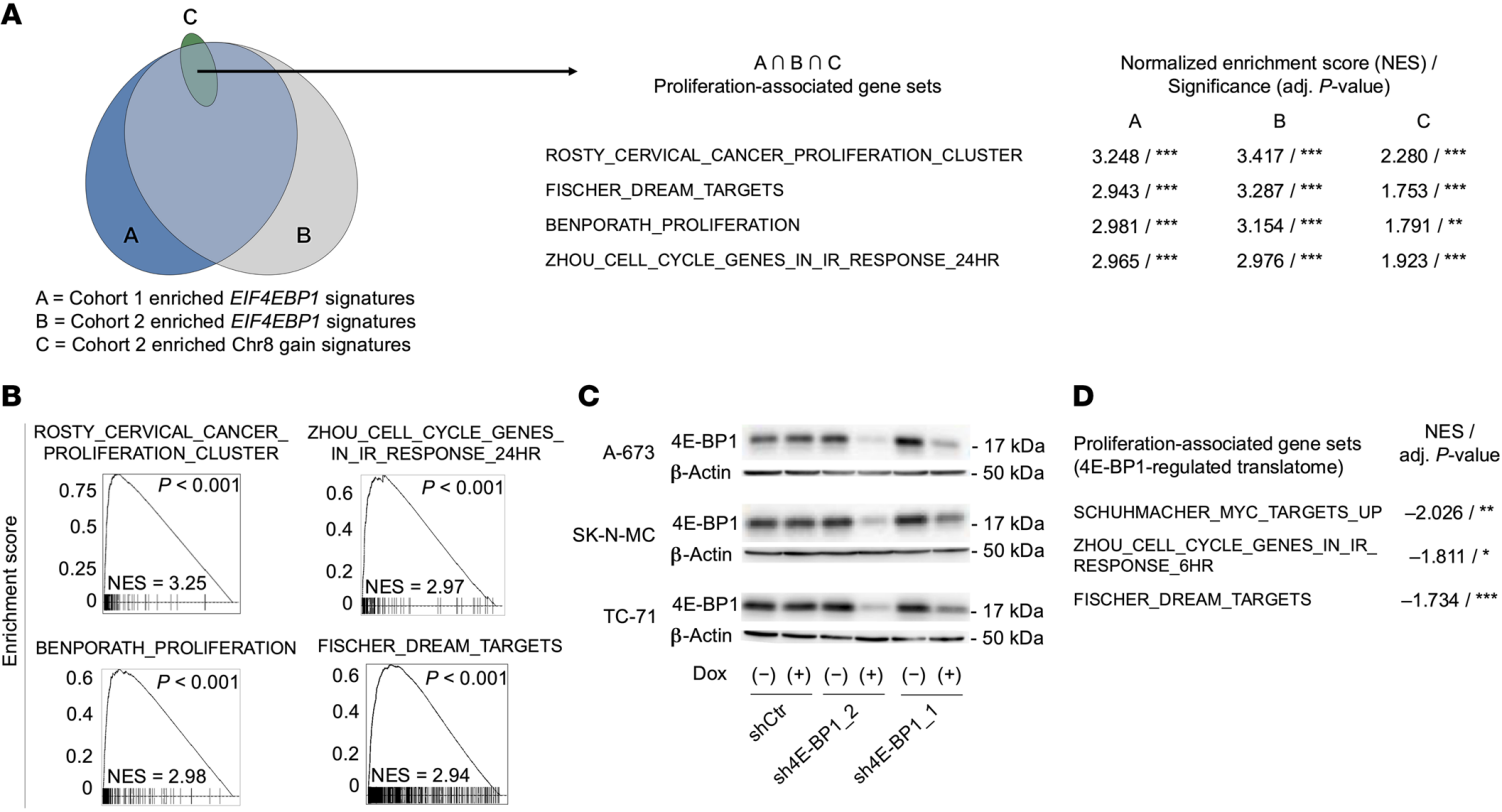
4E-BP1 drives a proliferation-associated proteomic network. (**A**) An area-proportional Venn diagram of gene sets enriched with *EIF4EBP1* expression in cohorts 1 (A) and 2 (B) as well as with chr8 gain in cohort 2 (C), as determined by fGSEA. Exemplary gene sets representing a proliferation-associated enrichment signature in the overlap among A, B, and C are shown with respective normalized enrichment scores (NESs) and significance levels. (**B**) fGSEA enrichment plots of exemplary gene sets displayed in (**A**). (**C**) Representative Western blots of A-673, SK-N-MC, and TC-71 cells containing either Dox-inducible specific shRNA constructs directed against *EIF4EBP1* (sh4E-BP1_1 or sh4E-BP1_2) or an shCtr. Cells were grown either with or without Dox for 96 hours. β-Actin served as a loading control. (**D**) Gene sets negatively enriched upon 4E-BP1 knockdown at the protein level, as determined by fGSEA using integrated MS and microarray protein/gene expression data as an input. Exemplary gene sets representing a proliferation-associated enrichment signature are shown with respective NESs and significance levels. ***P < 0.001, **P < 0.01, *P < 0.05. adj., adjusted.

Contrary to our findings that high *EIF4EBP1* levels significantly correlated with worse patient outcome (Figure 1, D, E, and H), a recent report has suggested that 4E-BP1 may act as a tumor suppressor in EwS (53). However, this conclusion was based on observations of supraphysiological, ectopic overexpression of a phospho-mutant (and thus functionally hyperactive) 4E-BP1 protein in 2 EwS cell lines (EW8 and TC-71) (53). The role of 4E-BP1 in cancer is complex and strongly depends on the cellular context and its precise phosphorylation status (30). Therefore, to obtain a more comprehensive understanding of 4E-BP1 in EwS, we first integrated results of preranked fast GSEA (fGSEA). We conducted fGSEAs based on Pearson’s correlation coefficients between the mRNA expression levels of *EIF4EBP1* and every other gene represented in the respective datasets of cohorts 1 and 2 (Supplemental Tables 5 and 6). Additionally, we carried out a third fGSEA based on gene expression fold-changes between tumors with and without detected chr8 gain in cohort 2 (Supplemental Table 7). The overlap among all 3 fGSEAs consisted predominantly of proliferation-associated gene sets (Figure 2, A and B, and Supplemental Tables 5–7). These transcriptomic data from patients with EwS pointed toward a role of 4E-BP1 in the regulation of EwS cell proliferation and strongly supported the potential role of 4E-BP1 as a major mediator of chr8 gain–driven poor prognosis in EwS.

To further explore this hypothesis, we generated an in vitro 4E-BP1 knockdown model in 3 EwS cell lines with relatively high *EIF4EBP1* baseline expression levels: A-673, SK-N-MC, and TC-71 (Supplemental Figure 2A). Notably, 2 of the selected cell lines (SK-N-MC and TC-71) exhibit a chr8 gain (26). Because chr8 gains are probably not the only factor affecting 4E-BP1 expression levels, and certainly not all chr8 gained tumors necessarily show high 4E-BP1 expression levels, we intentionally included 1 cell line (A-673) without chr8 amplification in the following analyses to emphasize the functional and clinical relevance of 4E-BP1 by itself across EwS with and without chr8 gain. Furthermore, A-673 cells were used because chr8 gain may affect expression levels of many other genes and thereby could bias the effects seen by modulation of 4E-BP1. To that end, we transduced these 3 EwS cell lines with a lentivirus containing a vector-system (pLKO Tet-on) with doxycycline (Dox)-inducible shRNAs specifically directed against *EIF4EBP1* (sh4E-BP1_1 or sh4E-BP1_2) or a nontargeting control shRNA (shCtr). Both targeted shRNAs effectively silenced *EIF4EBP1* mRNA expression, resulting in a strong knockdown of *EIF4EBP1* mRNA levels (Supplemental Figure 2B) and protein levels (Figure 2C and Supplemental Figure 2C). This is consistent with a strong correlation between (EIF)4E-BP1 mRNA and protein levels in human cells, as evidenced by the analysis of Cancer Dependency Map (DepMap) gene expression and corresponding protein array data (*n* = 887 cancer cell lines; *r* = 0.68; *P* = 5.2 × 10^–22^, by Pearson’s test). Western blot experiments demonstrated that knockdown of 4E-BP1 led to a consistent loss of its phosphorylated form (Ser65) in all EwS cell lines in a manner similar to total 4E-BP1 (Supplemental Figure 2, D and E). Therefore, our EwS 4E-BP1 knockdown models are well suited to study the functional consequences of its inactivation.

Because 4E-BP1 regulates mRNA translation initiation by binding to the translation initiation factor eIF4E and thereby modifies overall and selective translation rates (30), we asked whether functional interference with 4E-BP1 might also affect proliferation-related translational signatures. To identify proteins regulated by 4E-BP1 exclusively at the translational level, we combined mass spectrometry–based (MS-based) proteomic profiling of newly synthesized proteins (pulsed stable isotope labeled amino acids in cell culture [SILAC]) with parallel transcriptome profiling by gene expression microarrays. To this end, we silenced 4E-BP1 in the 3 aforementioned EwS cell lines and pulsed them with SILAC medium and the methionine analog l-azidohomoalanine for 6 hours. We identified 9,508 proteins through MS analysis, of which 4,335 common proteins across all cell lines and constructs with at least 1 value per replicate group were used for downstream analyses. Our parallel microarray analyses captured 12,056 stably expressed genes across all 3 cell lines. After more filtering steps (see Supplemental Methods), we identified 1,332 differentially expressed proteins upon 4E-BP1 knockdown (adjusted *P* < 0.05), which were not regulated by 4E-BP1 at the mRNA level across all 3 cell lines (Supplemental Table 8). To technically validate our MS findings, we conducted Western blot analyses for 1 representative upregulated protein, PDCD4 (54–57), after 4E-BP1 knockdown, thereby providing independent confirmation of our results using an alternative method (Supplemental Figure 2F).

Preranked fGSEA analysis on proteins not regulated at the mRNA level, and therefore most likely directly differentially regulated by 4E-BP1, identified again a strong enrichment of proliferation-associated gene sets (Figure 2D and Supplemental Table 9), consistent with fGSEA results from the patient gene expression data as shown in Figure 2, A and B. Such integrative fGSEA analyses conducted using the full list of obtained proteins and genes are displayed in Supplemental Tables 10 and 11, respectively, and essentially showed similar results.

Our in silico analyses of patient data at the mRNA level and functional in vitro analyses at the protein level collectively indicated 4E-BP1 is linked to accelerated proliferation of EwS cells, suggesting a potential role as an oncogene in EwS.

### 4E-BP1 promotes proliferation and tumorigenicity of EwS cells.

**Figure 3:**
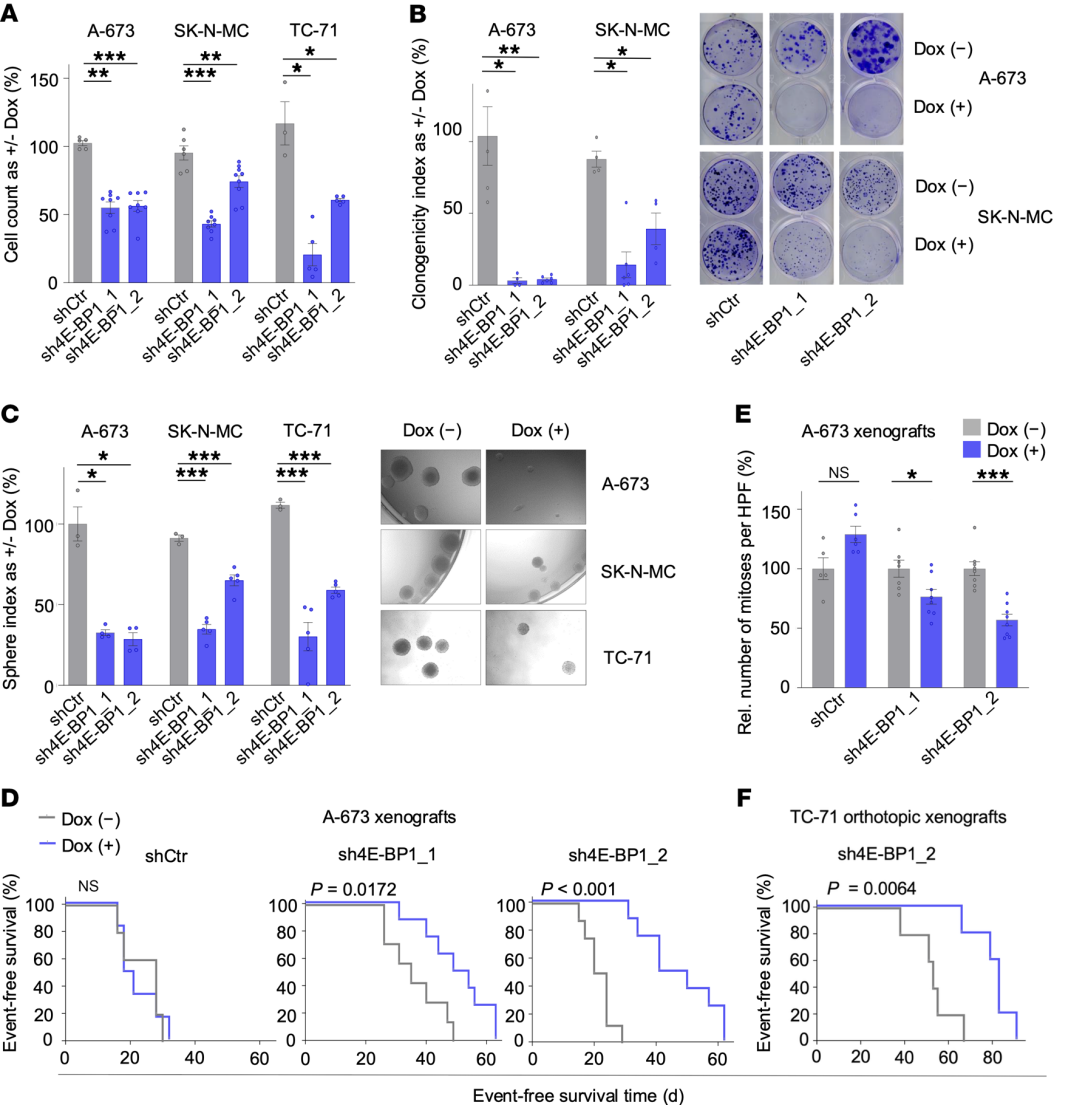
RNAi-mediated knockdown of 4E-BP1 inhibits EwS growth. (**A**) Relative viable cell count of A-673, SK-N-MC, and TC-71 cells containing either Dox-inducible specific shRNA constructs directed against *EIF4EBP1* (sh4E-BP1_1 or sh4E-BP1_2) or an shCtr, as measured by trypan blue exclusion. Cells were grown either with or without Dox for 120 hours. Horizontal bars represent means; whiskers represent the SEM; *n* ≥ 4 biologically independent experiments. *P* values were determined via 1-tailed Mann-Whitney test and adjusted for multiple comparisons with the Benjamini-Hochberg method. (**B**) Relative colony numbers of EwS cells containing indicated Dox-inducible shRNA constructs. Cells were grown either with or without Dox for 8–14 days. Horizontal bars represent means; whiskers the SEM; *n* ≥ 4 biologically independent experiments. *P* values were determined via 2-tailed Mann-Whitney test and adjusted for multiple comparisons with the Benjamini-Hochberg method. Representative images of colony formation are shown on the right. (**C**) Sphere formation in EwS cells containing indicated Dox-inducible shRNA constructs, treated with or without Dox for 8–14 days. Horizontal bars represent means; whiskers represent the SEM; *n* ≥ 3 biologically independent experiments. *P* values were determined by 2-tailed unpaired *t* test with Welch′s correction and adjusted for multiple comparisons with the Benjamini-Hochberg method. Representative images of spheres are shown on the right. (**D**) Kaplan-Meier analysis of event-free survival of NSG mice xenografted with A-673 cells containing indicated Dox-inducible shRNA constructs. Once tumors were palpable, mice were randomly assigned to treatment with either vehicle (–) or Dox (+); *n* ≥ 5 animals per condition. An “event” was recorded when tumors reached a size maximum of 15 mm in 1 dimension. *P* values were determined via Mantel-Haenszel test. (**E**) Quantification of mitoses in H&E-stained slides of xenografts described in (**D**). Five high-power fields (HPFs) were counted per sample. Horizontal bars represent means; whiskers represent the SEM; *n* ≥ 4 samples per condition. Rel, relative. (**F**) Kaplan-Meier analysis of event-free survival of NSG mice orthotopically xenografted into the proximal tibia with TC-71 cells containing a Dox-inducible shRNA construct directed against *EIF4EBP1*. *n* = 5 animals per condition. *P* values were determined via Mantel-Haenszel test. ***P < 0.001, **P < 0.01, *P < 0.05, determined via 2-tailed Mann-Whitney test if not otherwise specified.

To confirm the pro-proliferative and oncogenic roles of 4E-BP1 in EwS, we conducted various functional in vitro and in vivo assays. Knockdown of 4E-BP1 for 96 hours significantly inhibited cell proliferation in all 3 cell lines (Figure 3A). The antiproliferative effect of 4E-BP1 knockdown appeared to be independent of cell death, because trypan blue–exclusion assays did not consistently show a significant effect of 4E-BP1 knockdown on cell death across all cell lines and shRNAs (Supplemental Figure 3A). Prolonged 4E-BP1 knockdown (10–14 days) significantly reduced both 2D clonogenic and 3D anchorage-independent growth of EwS cells (Figure 3, B and C). Such effects were not observed in shCtr cells (Figure 3, B and C). Similarly, knockdown of 4E-BP1 in subcutaneously xenotransplanted cells significantly reduced tumor growth in vivo (Figure 3D, Supplemental Figure 3B, and Supplemental Table 12). Consistent with our in vitro results, this phenotype was linked to a significantly diminished mitotic cell count, as revealed by histologic assessment of the respective xenografts (Figure 3E and Supplemental Figure 3C). No difference in tumor necrosis was observed between xenografts with or without 4E-BP1 knockdown (Supplemental Figure 3D). Notably, combined MS and gene expression profiling of A-673 and TC-71 xenografts validated the proproliferative proteotranscriptomic signatures by fGSEA, as observed in vitro (Supplemental Tables 13 and 14).

To validate the effect of 4E-BP1 in an orthotopic xenograft model, we xenografted TC-71 cells transduced with an inducible *EIF4EBP1*-targeting shRNA construct (sh4E-BP1_2) into the proximal tibia of NSG mice, which were subsequently treated with or without Dox. Similar to our subcutaneous xenograft model, the tumor burden in orthotopic EwS xenografts decreased upon Dox-induced knockdown of 4E-BP1 (Figure 3F).

In summary, and in conjunction with our integrative clinical and in silico analyses of patient tumors and cell line models (Figures 1 and 2), these results generated in vitro and in vivo provide strong evidence that 4E-BP1 acts as an oncogene in EwS.

### High 4E-BP1 expression sensitizes for CDK4/6 inhibitor treatment.

**Figure 4:**
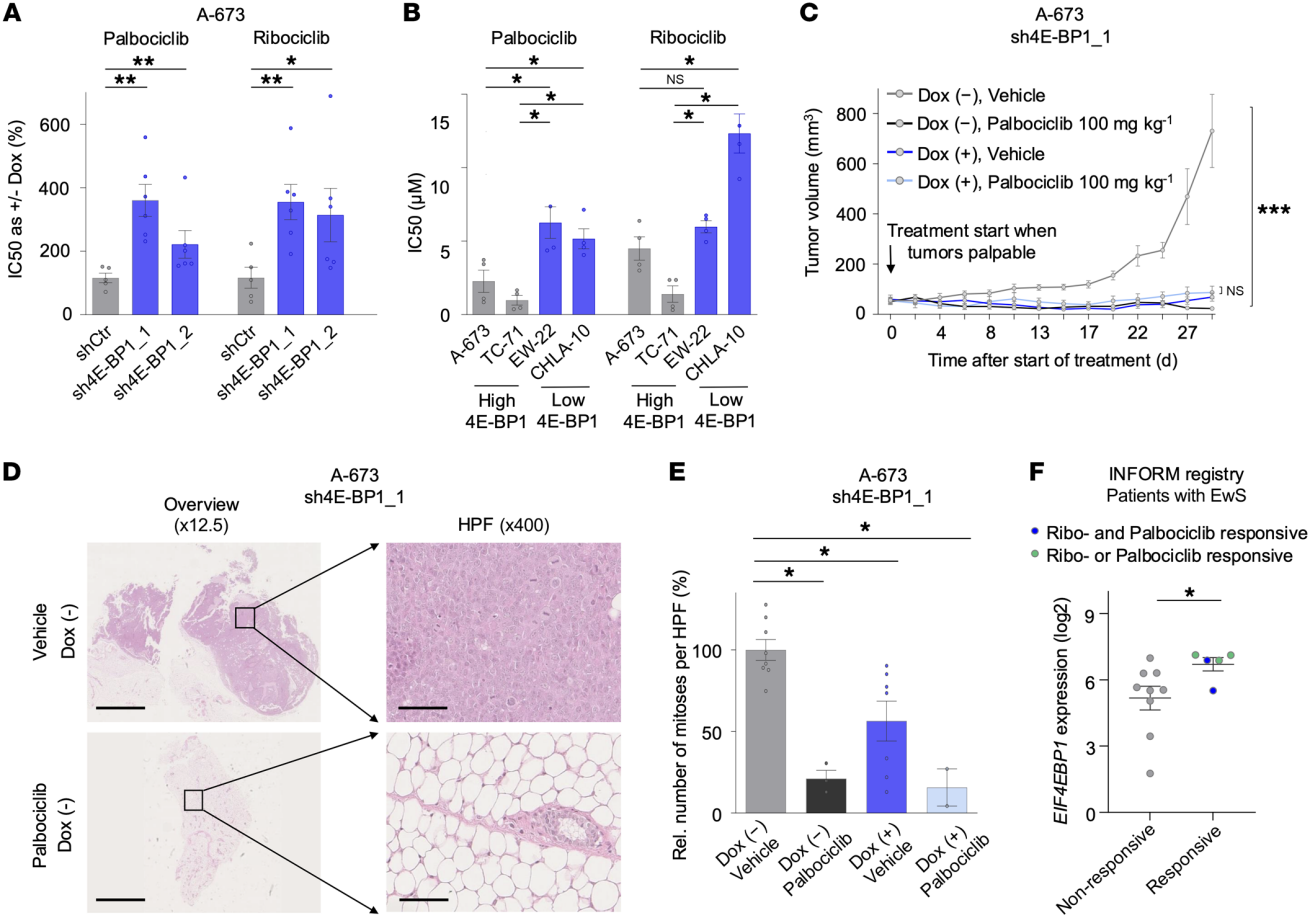
High 4E-BP1 expression sensitizes to targeted CDK4/6 inhibitor treatment with palbociclib and ribociclib. (**A**) IC_50_ analysis of CDK4/6 inhibitors palbociclib and ribociclib in A-673 cells containing indicated Dox-inducible shRNAs, as measured by resazurin colorimetry. Cells were treated with or without Dox as well as with serial dilutions of respective inhibitors. Horizontal bars represent means; whiskers represent the SEM; *n* ≥ 5 biologically independent experiments. *P* values were determined via 1-tailed Mann-Whitney test and adjusted for multiple comparisons with the Benjamini-Hochberg method. (**B**) IC_50_ analysis of CDK4/6 inhibitors palbociclib and ribociclib in EwS cells with high and low endogenous 4E-BP1 expression. Horizontal bars represent means; whiskers represent the SEM; *n* ≥ 3 biologically independent experiments. *P* values were determined via 1-tailed Mann-Whitney test and adjusted for multiple comparisons with the Benjamini-Hochberg method. (**C**) NSG mice xenografted with A-673 EwS cells containing a Dox-inducible sh4E-BP1 construct, treated with or without Dox and either vehicle or palbociclib. Mice were randomized to the treatment groups when tumors were palpable. For each condition, the mean tumor volume and SEM of 4–6 mice over the time of treatment are shown. *P* values were determined via 2-tailed Mann-Whitney test and adjusted for multiple comparisons with the Benjamini-Hochberg method. (**D**) Representative H&E-stained micrographs of A673/sh4E-BP1 xenografts [Dox (–)] treated with either vehicle or palbociclib, as described in (**C**) (shown as an overview with ×12.5 magnification and as a high-power field [HPF] at ×400 magnification). Scale bar: 2.5 mm (×12.5) and 100 μm (×400). (**E**) Quantification of mitoses in micrographs of xenografts described in (**C**). Horizontal bars represent means; whiskers represent the SEM; *n* ≥ 2 samples per condition. *P* values were determined via 2-tailed Mann-Whitney test and adjusted for multiple comparisons with the Benjamini-Hochberg method. Rel, relative. (**F**) *EIF4EBP1* gene expression data from EwS tumors of 14 patients treated within the INFORM registry and stratified according to matched palbociclib or ribociclib drug-sensitivity data from 3D tumor cell cultures into a CDK4/6 inhibitor nonresponsive and responsive groups. ***P < 0.001, **P < 0.01, *P < 0.05. *P* values were determined via 2-tailed Mann-Whitney test if not otherwise specified.

To identify therapeutic vulnerabilities in EwS with high 4E-BP1 expression, we conducted drug screens on 3D spheroids of A-673 EwS cells with or without knockdown of 4E-BP1. Ribociclib, an FDA-approved CDK4/6 inhibitor (58–61), was the top hit, demonstrating differential sensitivity in cells with high 4E-BP1 expression (Supplemental Figure 4A). The presented data align with the published gene-dependency data of the DepMap project, indicating a significant and selective dependency of EwS cell lines on CDK4 expression compared with non-EwS cell lines (Supplemental Figure 4B). We validated these findings in 2D culture experiments using A-673 EwS cells with or without 4E-BP1 knockdown and confirmed them in both A-673 and TC-71 EwS cells treated with the second FDA-approved CDK4/6 inhibitor, palbociclib (Figure 4A and Supplemental Figure 4C) (58–61). The top 3 hits identified in the drug screen after ribociclib (namely, vincristine, thioguanine, and methotrexate) exhibited no or comparatively lower increases in sensitivity upon 4E-BP1 knockdown in 2D experiments (Supplemental Figure 4D). Interestingly, EwS cell lines with high endogenous 4E-BP1 expression (A-673 and TC-71) showed greater sensitivity to palbociclib and ribociclib than did cell lines with low endogenous 4E-BP1 expression (EW-22 and CHLA-10) (Figure 4B and Supplemental Figure 4E). Consistently, overexpression of 4E-BP1 in an EwS cell line expressing 4E-BP1 at a low endogenous level (EW-22) led to an increase of sensitivity toward CDK4/6 inhibition (Supplemental Figure 4F).

Next, we conducted xenograft experiments by transplanting A673 EwS cells subcutaneously into the flanks of NSG mice, treated with or without Dox and with or without palbociclib; treatment started when tumors were palpable in all mice. Xenografts with 4E-BP1 knockdown and xenografts with palbociclib treatment similarly had a very strong reduction of tumor growth, which correlated with a strong decrease in histologically assessable viable tumor burden as compared with respective xenografts without 4E-BP1 knockdown or palbociclib treatment (Figures 4, C and D, Supplemental Figure 4G, and Supplemental Tables 15 and 16). Consistently, xenografts of mice with 4E-BP1 knockdown or treatment with palbociclib, for which histological material was obtainable, had fewer mitoses per high-power field (Figure 4E). However, because the very strong growth-inhibitory effects of either 4E-BP1 knockdown or palbociclib treatment alone precluded the assessment of a potential differential effect of 4E-BP1 expression on sensitivity toward palbociclib in this model (Figures 4, C and D, Supplemental Figure 4G, and Supplemental Tables 15 and 16), we turned to further validation to patient-derived real-world data. To this end, we analyzed gene expression and 3D drug sensitivity data from 14 short-term cultures treated with palbociclib or ribociclib from patients with EwS in the context of the Individualized Therapy for Relapsed Malignancies in Childhood (INFORM) registry (62). Strikingly, we found that high *EIF4EBP1* expression, indeed, was associated with higher sensitivity to CDK4/6 inhibitor treatment (Figure 4F).

To more mechanistically decipher the link between high 4E-BP1 expression, EwS cell proliferation, and increased sensitivity to CDK4/6 inhibitors, we screened the top 10% of downregulated proteins upon 4E-BP1 knockdown in our generated MS profiling (*n* = 393) according to a reported potential direct or indirect regulatory association with CDK4/6 signaling (*n* = 35). Furthermore, we screened for a significant association of the transcript expression of these genes with poor overall survival in cohort 1 (*n* = 11). Among the remaining 11 proteins, we further focused on those with potential mechanistic association with CDK4/6, according to the literature, leading to a final selection of 6 proteins: CDC25B (63), PRMT5 (64, 65), MCM2 (66, 67), RBL1 (68, 69), RNF2 (70, 71), and USP14 (72–74). To validate these results, we performed an association analysis with STRING (75), which showed a close association of most of these genes with 4E-BP1 and CDK4/6 (Supplemental Figure 4H). Furthermore, we performed complementary Western blot and parallel qRT-PCR assays measuring PRMT5 expression levels upon 4E-BP1 knockdown, showing that PRMT5 expression is reduced at the protein level but not at the mRNA level (Supplemental Figure 4I). These results validate our integrated pulsed SILAC and transcriptomic analyses, reinforcing the conclusion that the observed changes in protein abundance are primarily regulated at the level of protein synthesis.

We performed siRNA-mediated knockdown experiments for these genes in A-673 EwS cells and found that knockdown of CDC25B, PRMT5, and RBL1 significantly reduced cell proliferation without affecting cell death (Supplemental Figure 4, J–L); there was no significant effect on proliferation or cell death upon knockdown of the remaining 3 genes (data not shown). To validate these genes as critical mediators of 4E-BP1–related increased CDK4/6 inhibitor sensitivity, we performed 2D drug sensitivity assays and showed that knockdown of CDC25B and PRMT5 was associated with a reduction of sensitivity to CDK4/6 inhibition (Supplemental Figure 4M).

In summary, these results suggest 4E-BP1 may serve as a valuable predictive biomarker for clinical effectiveness of CDK4/6 inhibitor treatment.

## Discussion

The data presented herein from the EwS model establish chr8 gain as an unfavorable prognostic factor that primarily is mediated through the overexpression of 4E-BP1, which guides proproliferative proteomic signatures and sensitizes cells to targeted CDK4/6 inhibitor treatment.

In precision oncology, it is crucial to decipher mechanisms underlying inter-tumoral heterogeneity to refine diagnostic and therapeutic algorithms (14, 76, 77). In this context, the identification of chr8 gain as a prognostic factor emphasizes the relevance of cytogenetic testing, which may help stratify patients into prognostic and/or therapeutic subgroups. Although chr8 trisomies are observed in approximately 50% of patients with EwS, only trends or moderate associations between whole chr8 gain (i.e., trisomies) and poor patient outcome have been observed so far (21, 23–25, 51, 78, 79). Our data indicate there is a significant association between a high chr8 gene expression signature and poor overall survival of patients with EwS. Strikingly, this association was even more pronounced when exclusively considering patients without potentially confounding additional chromosomal gains and losses (Figure 1). Importantly, this association also remains statistically significant even when only considering patients with localized disease (Figure 1). Therefore, chr8 gain, as assessed by cytogenetic testing or FISH or a high chr8 gene expression signature score (i.e., as assessed by ssGSEA), might serve as a prognostic biomarker for poor overall patient survival. Consequently, it could be particularly useful for stratifying patients with localized disease into different treatment groups. Our results are consistent with those of studies from other cancer entities that have shown a prognostic/predictive value of chr8 gain (e.g., in acute myeloid leukemia, ref. 6; and chronic myeloid leukemia, refs. 4 and 5). Interestingly, chr8 gain is also observed in several other specific sarcoma entities, including myxoid liposarcoma (9), clear cell sarcoma (8), and pediatric undifferentiated sarcomas (10), as well as in several other cancer entities as shown in our analyses of TCGA data with partial prognostic value (Supplemental Table 4). Partial gains or losses of chr8 have been described in a broad range of cancer entities, such as prostate, lung, hepatocellular, and renal cell carcinomas (80–84). In contrast to the reported data from EwS, in some other types of cancer, chr8p losses are described to be associated with unfavorable clinical parameters (82, 84, 85). In the case of chr8q, gains are most frequently described as having tumor-promoting functions due to resulting *MYC* amplification (80, 83, 84, 86). However, in our patient cohort, a clinical association between *MYC* expression and overall survival was not evident (Supplemental Figure 1C and Supplemental Table 1), although MYC expression was linked to the expression of the proliferation marker Ki-67 and clinical outcome in EwS (87). Similarly, we did not find a significant association with overall patient survival for the chr8-located gene *RAD21* in our patient cohort (Supplemental Figure 1C and Supplemental Table 1), which was previously reported to promote EwS tumorigenicity by mitigating *EWSR1::FLI1–*induced replication stress (23). However, although we could not show a significant association of the expression of those genes with overall survival, it is conceivable that the biological effect of these genes is not necessarily linked to their mRNA abundance and is determined more by their absolute expression levels.

Our results show that poor survival outcomes associated with chr8 gains are primarily mediated by 4E-BP1 orchestrating a proproliferative proteomic network. However, the role of 4E-BP1 in cancer initiation or progression, especially whether 4E-BP1 exerts a protumorigenic or tumor-suppressing function, is still controversial, appears to be context dependent, and is not yet definite (30). 4E-BP1 has mostly been regarded as exerting tumor-suppressing functions by blocking cap-dependent translation or selective inhibition of specific transcript translation. Consistently, high levels of phosphorylated (and thus inactive) 4E-BP1 have been associated with poor outcome in many cancer entities (30, 43, 44). However, 4E-BP1 cannot be regarded as a bona fide tumor suppressor, because 4E-BP1 knockout mice did not develop tumors (88) and increasing evidence suggests the role of 4E-BP1 in cancer is more complex. In a context-dependent manner, 4E-BP1 can as well exert protumorigenic functions, such as promotion of hypoxia-induced angiogenesis and tumor formation in breast cancer (37) or conferring protection toward glucose starvation in glioma (42), both by selectively regulating translation of specific transcripts. Furthermore, 4E-BP1 is required for RAS-induced transformation in a p53-dependent manner (45). The data presented here in EwS are in favor of a tumor-promoting role of 4E-BP1, regulating a proproliferative proteomic network in EwS (Figures 2 and 3). This is consistent with our results showing a strong association of high *EIF4EBP1* expression levels with poor EwS survival (Figure 1). Such an association also is evident in numerous other cancer entities, as shown in our analysis of TCGA data (Supplemental Table 4), and is consistent with published deep computational analysis on pan-cancer TCGA data (46). However, the observed variances in 4E-BP1 mRNA and protein expression levels in patient tumors stratified by chr8 status (Figure 1, F and G) suggest chr8 gains may not be the only factor affecting 4E-BP1 expression levels in EwS. Apart from chr8 gains, other possible mechanisms of *EIF4EBP1* upregulation, such as direct upregulation driven by differential transcription-factor binding, have been described across cancer entities and also may account for high 4E-BP1 expression levels in individual tumors (14, 89).

We demonstrate here that high 4E-BP1 expression levels sensitize EwS cells to CDK4/6 inhibitor treatment with palbociclib and ribociclib (Figure 4). This effect may be mediated through direct translational regulation of CDC25B, a protein phosphatase critical for cell cycle progression and reported to contribute to tumorigenesis across multiple cancer types (63); and PRMT5, a methyltransferase that regulates diverse cellular processes, particularly transcription, and is similarly implicated in the progression of various malignancies (64, 65). Especially, PRMT5 is described to play an important role in mediation of CDK4/6 inhibitor sensitivity (64). Palbociclib and ribociclib are approved by the FDA for the treatment of hormone receptor–positive, EGFR2-negative advanced or metastatic breast cancer, used in combination with an aromatase inhibitor in postmenopausal women (58–61). In addition to their prognostic value in EwS (Figure 1), chr8 gain and, specifically, 4E-BP1 expression might serve as predictive markers to categorize patients with EwS into CDK4/6 inhibitor–sensitive and –nonsensitive groups. Such tailored stratification of patients into specific targeted treatment groups with drugs already approved by the FDA could significantly and promptly improve the outcomes of patients with EwS in the context of precision oncology. Notably, preclinical studies have already described a general sensitivity of EwS toward CDK4/6 inhibition, whereby IGF-1 receptor (IGF-1R) activation can mediated CDK4/6 inhibitor resistance (90, 91). As a result, a phase II clinical trial recently investigated palbociclib in combination with the IGF-1R inhibitor ganitumab for patients with relapsed or refractory EwS and reported a lack of adequate therapeutic activity, although a subgroup of patients had prolonged stable disease (92). However, that study did not include patient stratification based on predictive biomarkers. This gap might be addressed in future studies by incorporating predictive testing for chr8 gain and, especially, 4E-BP1 expression. Also, potential synergistic combination therapies might be needed to achieve full clinical effectiveness of CDK4/6 inhibition, which warrant further preclinical and clinical evaluation. The clinical importance of our findings is further highlighted by the currently ongoing Pfizer phase II trial testing treatment with irinotecan and temozolomide with or without palbociclib in patients with EwS (https://clinicaltrials.gov/study/NCT03709680) (93). However, the fact that high 4E-BP1 expression can be also observed in some tumor samples and cell lines without chr8 gains may hint that other factors may also contribute to its high expression and suggest that high 4E-BP1 expression may serve as a more robust predictive biomarker for response to CDK4/6 inhibitors than chr8 gains per se. Although our data suggest 4E-BP1 expression may, to some extent, enhance sensitivity to conventional chemotherapeutic agents, our primary focus was on the identification of targeted therapies whose efficacy is dependent on 4E-BP1 expression levels. This approach aims to inform the development of novel therapeutic strategies that could either reduce the adverse effects associated with standard chemotherapy or act synergistically with established treatment regimens in EwS therapy. Yet, it should be noted that chr8 gains may have broad functional effects that go beyond those of 4E-BP1 and its association with CDK4/6-sensitivity. Thus, CDK4/6 inhibition may only address part of the chr8 gain–mediated effects. Studies will need to dissect the other chr8-dependent phenotypes and how they crosslink with those mediated by 4E-BP1.

Collectively, our data suggest chr8 gain plays an important prognostic role in EwS and that its functional effects on tumor progression are primarily driven by increased 4E-BP1 expression mediating a proproliferative phenotype. Because chr8 gain occurs in approximately 50% of EwS cases, our results indicate this chromosomal aberration is a major source of intertumoral heterogeneity shaping the disease phenotype, clinical outcomes, and therapy options in EwS. Consequently, further cytogenetic testing of EwS might offer a refinement of clinical management within the context of precision oncology.

We establish chr8 gains and high 4E-BP1 expression as prognostic biomarkers in EwS and demonstrate that their association with patient outcome is primarily mediated by 4E-BP1 orchestrating a proproliferative proteomic network sensitizing EwS for CDK4/6-inhibitors. Because chr8 gains occur in approximately 50% of EwS cases, our results indicate this chromosomal aberration is a major source of intertumoral heterogeneity shaping the disease phenotype, clinical outcomes, and therapy options in EwS. Consequently, our data suggest testing for chr8 gains may improve risk stratification and therapeutic management in EwS and other cancers in the context of precision oncology.

## Methods

Please see the Supplemental Methods.

### Sex as a biological variable.

Male and female mice were used for in vivo experiments. Male and female patients were included in patient data analyses.

### Study approval.

Animal experiments were approved by the government of Upper Bavaria and North Baden and conducted in accordance with Animal Research: Reporting of In Vivo Experiments (ARRIVE) guidelines, recommendations of the European Community (86/609/EEC), and UK Coordinating Committee on Cancer Research guidelines for the welfare and use of animals in cancer research.

### Data availability.

Microarray data are publicly available in the Gene Expression Omnibus under accession numbers GSE294433 and GSE295817. MS datasets were uploaded to the PRIDE portal and are publicly accessible under the project accession code PXD065282. A Supporting Data Values file is provided.

## Author contributions

CMF and ACE performed functional in vitro and in vivo experiments, bioinformatic and histological analyses, and data analysis and interpretation; designed all figures; and contributed to writing manuscript. MFO performed functional experiments and contributed to shRNA design and lentiviral transduction of cell lines. KA performed the MS and analyzed MS data. JS and AY contributed to dataset curation. JL helped conduct in vivo experiments. TLBH, FHG, MMLK, EV, AKC, and FZ assisted with experimental procedures. RW cloned vectors. FW conducted histological analyses. SO, RI, and AB contributed to conducting in vivo experiments. JA, AS, WH, and UD provided clinical information. MS provided financial support and laboratory infrastructure. OW, IO, HP, and AL performed drug screens. SMP and GL provided biological and technical guidance. LRP conducted methylation arrays and performed bioinformatic analyses. JK provided financial support and laboratory infrastructure for the MS. FCA carried out functional in vitro and in vivo experiments. JM coordinated and supervised the study; provided biological and technical guidance; performed functional experiments; conducted bioinformatic and histological analyses; analyzed and interpreted all data; contributed to writing the manuscript; and provided financial support. TGPG designed, coordinated, and supervised the study; provided biological and technical guidance; analyzed and interpreted all data; contributed to writing the manuscript; and provided financial support and laboratory infrastructure. FS supervised and supported RNA-Seq analyses. All authors read and approved the final manuscript. The contributions of the first 2 authors to this work were highly comparable in significance. Given the similarity in the amount and importance of their contributions, it was not possible to differentiate qualitatively between them. Therefore, we have designated shared first authorship. CMF is listed in first position and ACE in second position to reflect a modest difference in the overall quantity of work performed.

## Funding support

This work is the result of NIH funding, in whole or in part, and is subject to the NIH Public Access Policy. Through acceptance of this federal funding, the NIH has been given a right to make the work publicly available in PubMed Central.

- German Cancer Aid grant DKH-70112257 to TGPG.
- Matthias-Lackas foundation to the laboratory of TGPG.
- Dr. Leopold und Carmen Ellinger Foundation.
- Gert & Susanna Mayer Foundation.
- DFG grant 458891500.
- German Cancer Aid grants DKH-7011411, DKH-70114278, and DKH-70115315.
- Dr. Rolf M. Schwiete Foundation.
- SMARCB1 Association.
- Ministry of Education and Research (A Systems Medicine Approach to Stratification of Cancer Recurrence [SMART-CARE].
- Heterogeneity, Evolution and Resistance of Sarcomas Driven by Fusion Genes in Adolescents and Young Adults [HEROES-AYA]).
- Barbara and Wilfried Mohr Foundation.
- European Union (European Research Council–funded CANCER-HARAKIRI project, grant 101122595).
- Ministry of Education and Research, HEROES-AYA grant 01KD2207B, to JM, IO, OW, MS, AB, and UD.
- Heidelberg Foundation of Surgery to JM
- Barbara und Wilfried Mohr Foundation to JM.
- China Scholarship Council to JL.
- German Cancer Aid and the German Academic Scholarship Foundation scholarships to CMF and ACE.
- German Cancer Aid scholarship to TLBH.
- Heinrich F.C. Behr Foundation scholarship to EV.
- German Cancer Aid grants DKH-108128 and DKH-70113419 to UD.
- Instituto de Salud Carlos III grants PI20CIII/00020, DTS22CIII/00003, and PMP21-00073 to the laboratory
- of JA.
- Fundación La Marató de TV3 grant 201937-30-31 to the laboratory of JA.
- Asociación Pablo Ugarte to the laboratory of JA.
- Fundación Sonrisa de Alex to the laboratory of JA.
- Asociación Todos somos Iván to the laboratory of JA.
- Asociación Candela Riera to the laboratory of JA.

## Supplementary Material

Supplemental data

Unedited blot and gel images

Supplemental tables 1-18

Supporting data values

## Version 1 10/16/2025

In-Press Preview

## Version 2 12/15/2025

Electronic publication
