# Supplementary material for: High 4E-BP1 expression associates with chromosome 8 gain and CDK4/6 sensitivity in Ewing sarcoma
Source: J Clin Invest. 2025 Oct 16;135(24):e187627. doi: 10.1172/JCI187627 (PMC12700544; doi:10.1172/JCI187627)
Supplement: Supplemental data [file jci-135-187627-s286.pdf]

SUPPLEMETARY INFORMATION

Funk & Ehlers et al.

High 4E-BP-1 expression associates with chromosome 8 gain and CDK4/6 sensitivity in Ewing Sarcoma

MATERIALS AND METHODS

Provenience of cell lines and cell culture conditions

Human EwS cell line A-673 (RRID: CVCL_0080) and human HEK293T cells (RRID: CVCL_0063) were purchased from American Type Culture Collection (ATCC). The human EwS cell line SK-N-MC (RRID: CVCL_0530) as well as HEK293T (RRID: CVCL_0063) cells were provided by the German collection of Microorganisms and Cell cultures (DSMZ). Human EwS cell lines CHLA-10 (RRID: CVCL_6583) and TC-71 (RRID: CVCL_2213) was provided by the Children's Oncology Group (COG). Human EW-22 (RRID: CVCL_1214) EwS cells were kindly provided by O. Delattre (Institut Curie, Paris, France). All cell lines were grown at 37 °C and 5% CO_2 in humidified atmosphere in RPMI 1640 medium supplemented with stable glutamine (Biochrom), 10% tetracycline-free fetal calf serum (FCS) (Sigma-Aldrich), 100 U/mL penicillin (Biochrom), and 100 µg/mL streptomycin (Biochrom) was used to grow the cells. Cells were routinely examined by nested PCR for mycoplasma infection. Cell line purity and authenticity was confirmed by STR-profiling.

Nucleic acid extraction, reverse transcription, and quantitative real-time PCR (qRT-PCR)

Genomic DNA from human cell lines was extracted with the NucleoSpin Tissue kit (Macherey-Nagel), plasmid DNA was extracted from bacteria with the PureYield kit (Promega). RNA was extracted with the NucleoSpin II kit (Macherey-Nagel) and reverse-transcribed using the High-Capacity cDNA Reverse Transcription kit (Applied Biosystems). qRT-PCR reactions were performed using SYBR green Mastermix (Applied Biosystems) mixed with diluted cDNA (1:10) and 0.5 µM forward and reverse primer (total reaction volume 15 µL) on a BioRad Opus instrument

and analyzed using BioRad CFX Manager 3.1 software. Gene expression values were calculated using the $2^{-(\Delta\Delta Ct)}$ method (1) relative to the housekeeping gene *RPLP0* as an internal control. Oligonucleotides were purchased from MWG Eurofins Genomics and are listed in **Supplementary Table 17.** The thermal conditions for qRT-PCR were as follows: initialization (95 °C, 2 min) (1 cycle); denaturation (95 °C, 10 sec), annealing (60 °C, 10 sec), and extension (60 °C, 10 sec) (49 cycles); denaturation (95 °C, 30 sec), annealing (65 °C, 30 sec), extension (melting curve 65 °C increasing 0.5 °C every 5 sec until 95 °C) (1 cycle).

Generation of doxycycline (Dox)-inducible shRNA constructs

Human EwS cell lines A-673, SK-N-MC, and TC-71 were transduced with lentiviral Tet-pLKO-puro all-in-one vector system (plasmid #21915, Addgene) containing a puromycin-resistance cassette, and a tet-responsive element for Dox-inducible expression of shRNAs against *EIF4EBP1* (sh4E-BP1_1 or sh4E-BP1_2) or a non-targeting control shRNA (shCtr). Sequences of the used shRNAs are listed in **Supplementary Table 17**. Dox-inducible vectors were generated according to a publicly available protocol (2) using In-Fusion HD Cloning Kit (Clontech). Vectors were amplified in Stellar Competent Cells (Clontech) and respective integrated shRNA was verified by Sanger sequencing. The used sequencing primer is listed in **Supplementary Table 17**. Lentiviral particles were generated in HEK293T cells. Virus-containing supernatant was collected to infect the human EwS cell lines. Successfully transduced cells were selected with 1.5 µg/mL puromycin (InvivoGen). The shRNA expression for *EIF4EBP1* knockdown or expression of a negative control shRNA in EwS cells was achieved by adding 0.5 µg/mL Dox every 48 h to the medium. Generated cell lines were designated as A-673/TR/shCtr, A-673/TR/sh4E-BP1_1, A-673/TR/sh4E-BP1_2, SK-N-MC/TR/shCtr, SK-N-MC/TR/sh4E-BP1_1, SK-N-MC/TR/sh4E-BP1_2, TC-71/TR/shCtr, TC-71/TR/sh4E-BP1_1, and TC-71/TR/sh4E-BP1_2.

Western blot

A-673, TC-71, and SK-N-MC cells were treated for 96h with Dox to induce the *EIF4EBP1* knockdown. A-673, TC-71, CHLA-10 and EW-22 wildtype and EW-22 4E-BP1_OE cells were seeded for 48h before protein extraction was started. Whole cellular protein was extracted with RIPA buffer (Serva electrophoresis) containing protease inhibitor cocktail and phosphatase inhibitor cocktail (Roche). Western blots were performed following routine protocols (3) and specific band detection was achieved by the use of rabbit monoclonal anti-4E-BP1 (clone Y329, 1:2,000, ab32024, Abcam), rabbit polyclonal anti-p-4E-BP1 antibody (1:1,000, Ser65, #9451, Cell Signaling), rabbit monoclonal anti-PDCD4 (1:1,000, #9535, Cell Signaling), rabbit monoclonal anti-PRMT5 (1:10,000, ab109451, Abcam), mouse monoclonal anti-ß-actin (1:5,000, A-5441, Sigma-Aldrich) and mouse monoclonal anti-ß-tubulin (1:10,000, 66240-1, Protein-Tech). Anti-rabbit IgG horseradish peroxidase coupled antibody (1:2,000, sc-516102, Santa Cruz Biotechnology) and anti-mouse IgG horseradish peroxidase coupled antibody (1:2,000, sc-2357, Santa Cruz Biotechnology) were used as secondary antibodies. Quantification of western blots was performed using ImageStudio Light software.

Proliferation assays

Depending on the cell line, 30–70 × 10^3 cells containing either a Dox-inducible non-targeting control shRNA or *EIF4EBP1*-targeting specific shRNAs were seeded in duplicate wells of a 6-well plate in 2 mL of growth medium. The cells were treated either with or without Dox (0.5 µg/mL; Sigma-Aldrich) for 120 h (medium was changed after 72 h including fresh Dox). Afterwards, cells of each treatment condition were harvested (including supernatant), stained with Trypan blue (Sigma-Aldrich), and viable as well as dead cells were counted in a standardized hemocytometer (C-chip, NanoEnTek). The assays were performed according to routine protocols (4). Sequences of shRNAs are listed in **Supplementary Table 17**.

For proliferation assays of siRNA-mediated knockdown of 4E-BP1 target genes, A-673 EwS wildtype cells were seeded in duplicate wells of a 6-well plate in 2 mL of growth medium at densities of 50 × 10^3 cells per well. 24 h after seeding cells were transfected with either a negative control siRNA pool or a siRNA pool directed against *CDC25B*, *PTRM5*, or *RBL1* according to manufacturer's instructions. Media was changed 4 h after transfection. After 120h (96h after transfection), cells of each treatment condition were collected and stained with Trypan blue. Viable and dead cells were counted in a standardized hemacytometer. siPools were customized and purchased from siTOOLs Biotech (Planegg/Martinsried, Germany) (Negative Control, Lot Nr: NegC-120.003; CDC25B, Lot-Nr: CDC25B-c1-001; PRMT5, Lot-Nr: PRMT5-003; RBL1, Lot-Nr: RBL1-002).

Clonogenic growth assays (Colony forming assays)

Depending on the cell line, 500–1,000 cells containing either a Dox-inducible non-targeting control shRNA or *EIF4EBP1*-targeting specific shRNAs were seeded in triplicate wells of a 12-well plate in 2 mL of growth medium. Cells were grown with or without Dox (0.5 µg/mL) for 8–14 d depending on the cell line and afterwards stained with crystal violet (Sigma-Aldrich). Colony number and area was determined on scanned plates using Fiji (Image J) (5, 6). Clonogenicity index was calculated by multiplying the counted colonies with the corresponding colony volume.

Spheroidal growth assays

Depending on the cell line, 1,500–4,000 cells containing either a Dox-inducible non-targeting control shRNA or *EIF4EBP1*-targeting specific shRNAs were seeded in Costar Ultra-low attachment plates (Corning) for 8–14 d in 150 µL growth medium. To maintain the 4E-BP1 knockdown, 10 µL of fresh medium with or without Dox was added every 48 h to the cells. At day 8–14, the wells were photographed and spheres larger than 500 µm in diameter were counted. The area was measured using ImageJ. The sphere volumes were calculated as follows: $V = 4/3 \times \pi \times r^3$.

The sphere index was calculated by multiplying the counted colonies with the corresponding colony volume. Quantification of sphere formation as shown in **Figure 3c** (left) has been performed on the entire well surface. The pictures shown in **Figure 3c** (right) should just serve as representative images reflecting precise quantification as shown in **Figure 3c** (left).

Generation of *EIF4EBP1* overexpression constructs

ORFeome collaboration cDNA clone IMAGE:100003200 encoding synthetic full-length open reading frame of human EIF4EBP1 gene coding sequence (CDS) (7) embedded in pENTR221 vector was obtained from the Vector and Clone Repository of the Genomics & Proteomics Core Facility (German Cancer Research Center (DKFZ), Heidelberg, Germany). pENTR221-EIF4EBP1 plasmid DNA was used as template to PCR-amplify EIF4EBP1 coding sequence using Platinum™ SuperFi II DNA Polymerase (Thermo Fisher Scientific) following the manufacturer's protocol; PCR reactions (20 µL) contained 1 ng of template DNA and 0.5 µM of each forward and reverse primer (see **Supplementary Table 16** for primer sequences). Extension was carried out at 60 °C for 15 s. The resulting PCR product was verified on a 1% agarose gel, yielding the expected ~360 bp band, and purified using the NucleoSpin® Gel and PCR Clean-up kit (Machery Nagel). The lentiviral backbone pLV-EF1a-IRES-Neo (Addgene #85139) was double-digested with BamHI-HF and EcoRI-HF endonucleases (NEB) in a 50 µL reaction at 37 °C for 1 h. Linearized digested vector was purified by agarose gel extraction using the NucleoSpin® Gel and PCR Clean-up kit (Machery Nagel). 2,5 ng of purified insert and 25 ng of linearized vector (2:1 molar ratio) were assembled using NEBuilder HiFi DNA Assembly Master Mix (NEB) in a 10 µL reaction at 50 °C for 15 min according to the manufacturer's protocol. Following assembly, 1 µL of the assembly mix was transformed into 50 µL NEB Stable High-Efficiency Competent E. coli (NEB) according to the manufacturer's protocol. Transformed cells recovered in 950 µL outgrowth medium (NEB) at 30 °C and 300 rpm for 1 h and were then plated on LB-agar supplemented with 100 µg/mL ampicillin (Sigma) and incubated overnight at 30 °C. One random colony was picked and expanded in a 100

mL LB culture supplemented with 100 µg/mL ampicillin at 30°C and 200 rpm overnight. Plasmid DNA was isolated using the ZymoPURE II Plasmid Maxiprep Kit (Zymo Research). Insert sequence was verified by Sanger sequencing using the EF1alpha sequencing primer (Microsynth Seqlab GmbH, Göttingen, Germany) and aligned to the reference EIF4EBP1 coding sequence using SnapGene (version 8.1) to confirm the absence of mutations. Lentiviral particles were generated in early-passage HEK293T cells using psPAX2 (Plasmid #12260, Addgene) and pMD2.G (Plasmid #12259, Addgene) as packaging plasmids and the newly established pLV-EF1a-EIF4EBP1-IRES-Neo or pLV-EF1a-IRES-Neo as transfer plasmid or as control construct. HEK293T cells were transfected using PEI MAX transfection reagent (Polysciences). 10 mL of virus-containing supernatant was collected and filtered 72 h after transfection to infect the human Ewing sarcoma cell line EW-22. Successfully transduced cells were initially selected with 250 µg/mL Neomycin for 120 h (Sigma-Aldrich) and reselected with Neomycin after 2 weeks to enrich transduced cells. Successful ectopic expression of 4E-BP1 was confirmed at the protein level by western blotting using a specific antibody against 4E-BP1 (see section 'Western blotting').

Drug screening assays (3D)

Drug screening experiments were performed essentially as previously described in Peterziel et al. (8) using 384-well round bottom ultralow attachment spheroid microplates (# 3830, Corning) to allow the formation of three-dimensional spheroids.

Briefly, we tested a drug library of 75 drugs, mostly approved or in clinical trials, covering standard chemotherapeutic drugs, epigenetic modifiers, metabolic modifiers, kinase inhibitors, apoptotic modulators, and others (8–10). Drug plates were ordered from FIMM High Throughput Biomedicine Unit (Institute for Molecular Medicine Finland HiLIFE, University of Helsinki, Finland) as ready-to-use assay plates and stored in an oxygen- and moisture-free environment (San Francisco StoragePod, Roylan Developments Ltd, Fetcham Leatherhead, UK) at room temperature until use. One drug plate set consisted of three plates. The concentration range of each drug covered five

orders of magnitude with each condition tested in duplicate. Wells containing 100 µM benzethonium chloride (BztCl), 250 nM staurosporine (STS) (serving as a death control), and 0.1% DMSO were included as maximum, intermediate, and minimum effect controls, respectively. An STS concentration range of 0.1 to 1,000 nM served as a technical control (two replicates per plate). Per well, 25 µL of single-cell suspension was dispensed on ready-to-use plates with an 8-channel electronic Picus pipette (10 to 300 µL, #735361, Sartorius).

As a read-out for cell viability, bulk ATP quantitation with CellTiter-Glo® 2.0 (CTG; #G9243, Promega) to determine the relative number of metabolically active cells per well was performed 72 h after treatment according to the manufacturer´s protocol.

Prior to the drug screen, the suitable cell number to be seeded per well was determined by assessing the proportionality between the number of cells and the luminescence signal for 200, 500, 1,000 and 2,000 cells in the absence of drug treatment. The cell numbers used for the subsequent screening experiment were 250 cells per well for A-673 Dox (–) and 350 cells per well for A-673 Dox (+).

Drug effects were calculated as drug sensitivity scores (DSSasym) using the web-based drug analysis pipeline iTReX(10).

In analyses of Ribociclib and Palbociclib drug sensitivity screenings of primary patient cultures as determined in the context of the INFORM registry (11), a culture was considered as responsive if a drug sensitivity score ranged in the highest quartile, whereas samples ranging in the lower 3 quartiles were considered as non-responsive.

CDK4/6 inhibitor assays in vitro

A-673 and TC-71 (high endogenous 4E-BP1 expression) as well as CHLA-10 and EW-22 (low endogenous 4E-BP1 expression) EwS cells were seeded in a 96-well plate at a density of 0.5 –2 × 10^3 per well. In case of cells containing Dox-inducible constructs, cells were treated with/without Dox (0.5 µg/mL; Sigma-Aldrich). 48 h after seeding or pre-incubation with Dox, respectively, CDK4/6 inhibitors (Palbociclib or Ribociclib; Biozol Diagnostica and Hölzel Diagnostika) were

added in serially diluted concentrations ranging from 0.0032 to 500 µM. Each well contained an equal concentration of 0.5% DMSO (Sigma-Aldrich). Cells only treated with 0.5% of DMSO served as a control. After 72 h of inhibitor treatment, the plates were assayed on a GloMax® Explorer Multimode Microplate Reader after incubation with Resazurin (16 µg/mL; Sigma-Aldrich) for 4-6 h.

For differential sensitivity assays upon 4E-BP1 overexpression, EW-22 cells transduced with a negative control and EW-22 cells transduced with a 4E-BP1 overexpression vector were seeded in a 96-well plate at a density of 4×10^3 cells per well. 24 h after seeding Ribociclib was added in serially diluted concentrations (0.64 nM to 50 µM). 72 h after addition of Ribociclib, plates were incubated with resazurin (16 µg/mL) for 4–6 h and assayed on a microplate reader.

For differential sensitivity assays upon CDC25B and PRMT5 knockdown, A-673 EwS wildtype cells were seeded in a 96-well plate at a density of 0.9×10^3 cells per well. 24 h after seeding cells were transfected with either a Control siRNA or a siRNA directed against *CDC25B* or *PRMT5* according to manufacturer's instructions. 48 h after siRNA transfection Ribociclib was added in serially diluted concentrations (0.64 nM to 50 µM). 72 h after addition of Ribociclib, plates were incubated with resazurin (16 µg/mL) for 4–6 h and assayed on a microplate reader.

Mass spectrometry (MS)

Pulse-Chase Labelling using AHA (azidohomoalanine) and SILAC isotope labels for nascent proteome analysis: A-673, SK-N-MC, and TC-71 EwS cells containing either a Dox-inducible non-targeting control shRNA or *EIF4EBP1*-targeting specific shRNAs (sh_4E-BP1_1 or sh_4E-BP1_2) were seeded at a density of 1×10^6 per 15-cm dish in 20 mL of culture medium and grown with or without Dox (0.5 µg/mL; Sigma-Aldrich) for 96h. Subsequently, cells were washed with pre-warmed PBS and cultivated in 10 mL SILAC RPMI deprivation media (lysine-, arginine-, and methionine-free) (Athenas) for 45 min at 37 °C. This ensures the depletion of the intracellular stores of amino acid. The depletion medium was then aspirated and cells were incubated in 13 mLof

SILAC RPMI pulse media containing 18.1 mg/L AHA (Jena Bioscience), and either 200 mg/L [13C6, 15N4] L-arginine and 40 mg/L [13C6, 15N2] L-lysine or 200 mg/l [13C6] L-arginine and 40 mg/L [4,4,5,5-D4] L-lysine (Silantes) for 6h at 37°C with 5% CO_2. Following pulse labelling, cells were washed with PBS and harvested by scraping and centrifugation for 5 min at 400× g. Cell pellets were frozen and stored at –80°C until lysis. Enrichment of newly synthesized proteins for translatome analysis and sample preparation for LC-MS/MS measurements: Newly synthesized proteins were enriched using the Click-It alkyne agarose enrichment kit (Thermo Fisher) according to the manufacturer protocol with minor modifications. Cell pellets were lysed in 900 µL of Urea Lysis buffer (200 mM HEPES pH8, 0.5 M NaCl, 4% CHAPS, 8 M Urea), and cell lysates were sonicated on ice using the Benson probe sonicator. Protein concentrations were determined using the BSA kit (Thermo Fisher) and 1 mg of each label pair (+/– Dox) were mixed in a new tube. Volumes were made up to 1,700 µL end volume with 8M Urea lysis buffer. Next, the mixed lysates were combined with 100 µL of the alkyne-agarose slurry and 93 µL of the [3+2] cyclo-addition reaction mixture (10 µL Cu(II)SO4 (200 mM), 62.5 µL tris (hydroxypropyltriazolylmethyl) amine (THPTA, 160 mM), 10 µL aminoguanidine (2 M), 10 µL sodium ascorbate (2 M)), and incubated on a ThermoMixer for 2 h at 40°C. Following the incubation step, the resins were pelleted by centrifugation for 1 min at 2,000× *g* and supernatants were discarded. Subsequently, the resins were washed once with 2 mL of MQ water and pelleted by another centrifugation step. Next, the resins were resuspended in 2 mL of the 1-step reduction/alkylation mixture (10 mM Tris(2-carboxyethyl)phosphine, 40 mM 2-Chloroacetamide in SDS wash buffer) and incubated on a ThermoMixer for 15 min at 70 °C and another 15 min at 20 °C. Following the reduction/alkylation step, the resins were transferred into spin columns (BioRad) and placed on a P10 tip-box for the subsequent steps. The resins were consecutively washed five times with 1 mL SDS-Wash buffer (100 mM Tris-HCl pH 8.0, 1% SDS, 250 mM NaCl, 5 mM EDTA), once with MQ water, five times with Guanidine-Wash buffer (100 mM Tris-HCl pH 8.0, 6M Guanidine-HCl), and 5× with Acetonitrile-Wash buffer (20% Acetonitrile, ULCMS in Water ULCMS). Subsequently, the resins

were resuspended in 200 µL Digestion buffer (100 mM Tris-HCl pH 8, 2 mM CaCl2, 5% Acetonitrile), transferred to new tubes, and subjected to proteolytic digestion by adding 1 µg of trypsin on a ThermoMixer for 16 h at 37 °C. Following the digestion step, the resins were pelleted by centrifugation for 5 min at 2,000× g, the supernatants containing the digested peptides were transferred to new tubes and acidified with formic acid (FA) to 1% end concentration. For the peptide clean-up step, Oasis PRiME HLB µElution Plates (Waters) were used according to the manufacturer protocol. Briefly, the digested peptides were transferred to the Oasis plate and washed consecutively with 750 µL, 250 µL, and 100 µL of 1% FA. Peptides were eluted in a 96-well plate by adding 70 µL of elution solution (60% MeOH, 1% FA, 39% Water ULCMS) and another 50 µL of 100% MeOH. The eluted peptides were then dried down using a SpeedVac, resuspended in 0.1% TFA, and samples were subjected to HPLC fractionation and mass spectrometry analysis. LC-MS/MS analysis: Samples were analyzed on an Orbitrap Fusion mass spectrometer (Thermo Fisher) coupled with an Easy-nLC 1200 system (Thermo Fisher). Chromatographic separation was carried out using an Acclaim Pepmap RSLC trap column (100 µm × 2 cm, 5 µm particles, 100 Å pores, C18, Thermo Fisher), and nanoEase M/Z Peptide BEH analytical column, (75 µm × 250 mm, 1.7 µm particles, 130Å pores, C18, Waters). Peptides elution gradient was set to 105 min at flow rate of 300 nL/min using solvent A (0.1% formic acid in ULCM grade water) and solvent B (0.1% formic acid in 80% acetonitrile and 19.9% ULCM grade water). Samples were injected into the mass spectrometer using a 10 µm Picotip coated fused silica emitter (New Objective). The Orbitrap-Fusion mass spectrometer was operating in positive mode. Acquisition was carried out in data-dependent acquisition (DDA) mode. The MS1 scan was detected in orbitrap mode at 60,000 FWHM resolution, AGC target 1E6, scan range (m/z) was 375–1,500 DA, and maximal injection time was set to 50 ms. The intensity threshold HCD-fragmentation was set to 5E3. MS2 detection was acquired in centroid mode with an HCD collision energy of 33% in an ion trap detector, an isolation window (m/z) of 1.6 Da, AGC target 1E4, and maximal injection time of 50 ms. Data analysis: The raw files were analyzed using MaxQuant (version 1.6.10.43). The MS/MS spectra were searched

through the integrated Andromeda search engine against Homo Sapiens UniProt proteome database. Multiplicity of labels was set to 3. Arg6/Lys4 and Arg10/Lys8 were selected as medium and heavy labels, respectively. Cysteine carbamidomethylation was selected for fixed modification. Methionine oxidation, protein N-terminal acetylation, replacement of methionine by AHA and conversion of AHA to homoserine (HS) and diaminobutyrate (DAB) were selected for variable modifications. Re-quantify and match between runs (match time window: 0.4 min) functions were enabled. For the digestion, Trypsin/P was selected, and the maximal number of mis-cleavages was set to 2. The minimal peptide length was set to 7 amino acids and FDR for peptide and protein identification was set to 0.01. For protein identification, at least one unique peptide was required. Minimum ratio count for label-based protein quantification was set to 2. Normalized H/M ratios derived from the ProteinGroups file were further processed and subjected to statistical analysis. FDR was set to >0.05 and S0 to 0.1. MS profiling was performed in biological triplicates for every cell line/construct/condition. This process identified 9,508 proteins through MS analysis, of which 4,335 common proteins across all cell lines and constructs with at least one value per replicate group were used for downstream analyses.

For proteomic analysis from xenografts, FFPE samples were homogenized for 20 min in 75 µL SDS lysis buffer (4% SDS, 100 mmol/L Ammonium bicarbonate, pH 8.5) using the Beatbox (PreOmics). For protein de-crosslinking, samples were boiled for 1 h at 95 °C. Next, samples were subjected to the AFA-ultrasonication using LE220R-plus ultrasonicator (Covaris). For protein extraction and purification 12 µL from each FFPE tissue lysate were used as a direct input for the autoSP3 protocol. The autoSP3 protocol, including protein clean-up, reduction and alkylation (using 10 mmol/L TCEP and 40 mol/L CAA at final concentration), and digestion (trypsin:protein ratio of 1:20), were performed on the Bravo liquid handling system (Agilent Technologies) as previously described (12, 13). Equivalent amount of 200 ng peptides per sample was injected into the timsTOF Pro mass spectrometer (Bruker Daltonics) coupled to an Easy nLC 1200 system (Thermo Scientific). The elution gradient was set to 80 min at a flow rate of 300 nL/min using solvent A (0.1% formic acid

in ULCM grade water) and solvent B (0.1% formic acid in 80% acetonitrile and 19.9% ULCM grade water). Data were acquired in DIA-PASEF mode. The full scan MS spectra were set to a mass range 100 to 1,700 m/z and 1/k0 range from 0.65 to 1.42 V*s/cm2 with a 100 ms ramp time. The duty cycle was locked at 100%, the ion polarity was set to positive, and TIMS mode was enabled. Collision energy was set to 1/k0 range from 0.65 to 1.42 V*s/cm2. For the DIA scans, a custom isolation window pattern was optimized, covering the precursor range of 377 to 1,194 m/z, mobility range 1/k0 range from 0.67 to 1.39 V*s/cm2, and cycle time estimate of 1.58 s. For data analysis, raw files were analyzed using DIA-NN 2.0.1 in library-free mode using the default settings. The protein database used for the search was the Homo sapiens (Taxon ID: 9606) and Mus musculus (Taxon ID: 10090) reviewed Swiss-Prot databases. In brief, FASTA digest for library-free search and deep learning –based spectra RTs and IMs prediction were enabled. Cysteine carbamidomethylation was enabled as a fixed modification. Maximum number of variable modifications was set to 1. For digestion, Trypsin/P was selected, and the maximal number of missed cleavages was set to 2. Minimum and maximum peptide length were set to 7 and 30, respectively. Minimum and maximum precursor charge were set to 1 and 4, respectively. The match-between-runs (MBR) function was allowed. Raw files were processed with DIA-NN software (version 2.0.1.) Human and mouse proteins were separated based on species-specific annotations, and low-quality samples (n=2) were excluded after quality control assessment. Data were log2-transformed, filtered to retain proteins detected in ≥50% of samples per group, and missing values imputed using the MinProb algorithm. Differential protein expression analysis was performed using the limma package (version 3.62.2).

For analysis of differential protein expression, $\log_2$ fold changes ($\log_2$FCs) between conditions with and without Dox treatment were calculated for every cell line, every construct, and every biological replicate. For every cell line/construct log2FCs were normalized to its respective shCtr. Next, a global median $\log_2$FC across all biological replicates was calculated for every protein. Proteins were only considered as being directly regulated by 4E-BP1 when their mRNA $|\log_2 \text{FCs}|$ were <0.5.

MS datasets were uploaded at the PRIDE portal and are publicly accessible under the project accession code PXD065282.

Xenotransplantation experiments

To assess local tumor growth of subcutaneous xenografts *in vivo*, 2.5 × 10^6 A-673 or TC-71 EwS cells containing either a Dox-inducible negative control shRNA or specific shRNAs against *EIF4EBP1* were injected subcutaneously with a 1:1 mix of PBS (Biochrom) and Geltrex (LDEV-Free Reduced Growth Factor Basement Membrane Matrix, Thermo Fisher Scientific; max volume 100 µL) in the right flanks of 4–8 week old NSG mice following routine protocols (14). When tumors were first palpable, mice were randomized to the control group (17.5 mg/mL sucrose (Sigma-Aldrich) in drinking water) or the treatment group (2 mg/mL Dox Beladox, bela-pharm) and 50 mg/mL sucrose (Sigma-Aldrich) in drinking water. According to the average water intake of mice in the different treatment groups, the concentration of sucrose in the different treatment groups has been adapted: due to the bitter taste of Dox, the mice that receive Dox via their drinking water in average have a lower water intake, making a higher concentration of sucrose necessary to ensure equal intake of sucrose per mouse across the different treatment groups. Tumor size was measured with a calliper every two days and tumor volume was calculated as $V=a \times b^2/2$ with *a* being the largest diameter and *b* the smallest. Right before tumors reached the maximum size of 15 mm in one dimension (event), the respective mice were sacrificed by cervical dislocation. Other specific humane endpoints were determined as follows: invasive tumor growth leading to functional impairment or pain, ulcerating tumor (or fluid externalization), prolonged obstipation, abdominal distention, peritonitis, ascites, bloody diarrhea, palpable abdominal tumor mass with additional signs of pain, total relief, or medium paresis of a limb. General humane endpoints were determined as follows: Loss of 20% body weight, apathy, piloerection, self-isolation, aggressivity, automutilation, unphysiological or reduced movements/positioning, abnormal breathing, and reaching of a maximum observation period of 12 months.

For analysis of EwS xenograft growth in bone, TC-71/TR/sh4E-BP1_2 EwS cells were orthotopically injected into the proximal tibial plateau of NSG mice. One day before injection, mice were pre-treated with 800 mg/kg mouse weight/d Metamizole in drinking water as analgesia. On the day of injection, mice were anesthetized with inhaled isoflurane (2.5% in volume) and their eyes were protected with Bepanthen eye cream. After disinfection of the injection site, 2×10^5 cells/20 µL were directly injected with a 30 G needle (Hamilton) into the right proximal tibia. For pain prophylaxis after intraosseous injection, mice were treated with Metamizole in drinking water (800 mg/kg mouse weight/d). The first day after injection of tumor cells, mice were randomized in two groups of which one received henceforth 2 mg/mL Dox (BelaDox, Bela-pharm) dissolved in drinking water containing 5% sucrose (Sigma-Aldrich) to induce sh4E-BP1_2 expression, whereas the other group only received 5% sucrose. All mice were closely monitored routinely every two days and tumor growth was evaluated with a caliper. All tumor-bearing mice were sacrificed by cervical dislocation when the mice exhibited first signs of limping at the injected leg (event) or reached any humane endpoint as listed above.

Xenograft growth is presented as Kaplan-Meier plots illustrating event-free survival, where the definition of an event varies according to the specific experiment (15–19). In this design, event-free survival serves as the primary endpoint, determined by the time at which tumors reach a predefined volume threshold. As a result, mice were typically sacrificed once tumors attained comparable sizes, leading to similar tumor volumes at the time of collection.

For CDK4/6 inhibitor treatment *in vivo*, A-673/TR/sh4E-BP1_1 cells were injected subcutaneously as described above. As soon as the tumors were palpable, mice were subjected to either the vehicle (DSMO) or the treatment group (Palbociclib, LC Laboratories, 100 mg/kg), whereby each group was treated with or without addition of Dox to the drinking water (Beladox, bela-pharm, 2 mg/mL). Palbociclib was administered by oral gavage for 28 days, with a break of 2 days every 5 days of treatment. The experimental endpoint was predetermined as 28 days after first injection of either inhibitor, or if humane endpoints as described above were reached before. To examine the number

of mitoses within the tumors, hematoxylin and eosin (HE) stained slides of the respective tumors were examined and the number of mitoses were quantified as described below in section 'Histology'. Animal experiments were approved by the government of Upper Bavaria and North Baden and conducted in accordance with ARRIVE guidelines, recommendations of the European Community (86/609/EEC), and United Kingdom Coordinating Committee on Cancer Research (UKCCCR) guidelines for the welfare and use of animals in cancer research.

Male and female mice were used for the experiments.

Methylation arrays and CNV analysis

A minimum of 500 ng of high-quality DNA from 100 EwS FFPE samples was used for methylation and CNV analysis with the Infinium Human Methylation 450K BeadChip (EPIC array, Illumina). This method can analyze 864,928 CpGs including main CpG islands and CpG sites outside of CpG islands. Raw methylation and CNV data were essentially processed as previously reported (20). Based on the methylome, we used a previously described sarcoma classifier (20), which assigns a score to each sample indicating the similarity of the respective methylome with methylation patterns of samples from known entities. CNVs were also assessed by this method and analyzed at both, chromosomal and specific locus level. Scores for specific positions were visualized in the Integrative Genomic Viewer (IGV).

For analysis regarding the association between chr8 gain and *EIF4EBP1* expression (as assessed by RNA-seq), only samples were analyzed which were clearly possible to separate into either a group showing global chr8 gain or no chr8 gain. Samples with partial gain were not considered.

For determining chr8 CNA status in tumor entities other than EwS we analyzed TCGA SNP array 6.0 pre-segmented data using TCGAbiolinks R package(21) (Masked Copy Number segment, GRCh38.p0). For downstream analysis we selected all primary tumor samples for which clinical annotation was complete and SNP array and RNA-seq datasets existed, we applied re-segmentation algorithm by CNApp (22) to classify samples as chr8 gain or no gain. Classification criteria for

whole chr8 gain were log2 segment mean > 0.2 and the longest segment spanning ≥ 75% of total chr8 length (chr8q length 68%). To compare both groups (chr8 gain vs. no gain) regarding overall patient survival we performed a Kaplan Meier analysis using the survival R package(23, 24). Significance levels were calculated using the log-rank test.

Male and female patients were included in the analyses.

RNA sequencing (RNA-seq)

RNA quality was assessed by the Agilent 2100 Bioanalyzer (Agilent Technologies) before library preparation. 2 µg of estimated high-quality RNA was prepared for sequencing. Libraries were prepared according to the TruSeq RNA Exome (Illumina) protocol. Library preparation workflow included purification and fragmentation of mRNA, first and second strand cDNA synthesis, end repair, 3´ends adenylation, adapters ligation, PCR amplification, library quantification, normalization and libraries pooling. Cleanup cycles are introduced between the mentioned steps. Sequencing was performed on a NextSeq 550 / NovaSeq 600 Sequencer (Illumina). NextSeq 550 was performed with 75 bp ‘paired-end’ sequencing technology employing high-output flow cells. Calculations of RNA counts were performed as previously described (25, 26).

Gene expression microarrays

A-673, TC-71, and SK-N-MC EwS cells containing either a Dox-inducible non-targeting control shRNA (shCtr) or *EIF4EBP1*-targeting specific shRNAs (sh4E-BP1_1 or sh4E-BP1_2) were seeded in T25 flasks (TPP) and treated either with or without Dox (0.5 µg/mL; Sigma-Aldrich) for 96 h. Thereafter, total RNA was extracted from one biological replicate for each condition with the Nucleospin II kit from Macherey-Nagel and transcriptome profiled at IMGM laboratories (Martinsried, Germany). Total RNA of A-673 and TC-71 xenografts, containing either a Dox-inducible non-targeting control shRNA (shCtr) or a *EIF4EBP1*-targeting specific shRNA (sh4E-BP1_2), that were injected into the right flanks of NGS mice and treated as described in section

'Xenograft experiments', was extracted from at least 5–8 biological replicates depending on the condition with the Nucleospin II kit from Macherey-Nagel and transcriptome profiled at Microarray core facility of the German Cancer Research Center Heidelberg.

RNA quality was assessed with a Bioanalyzer and samples with RNA integrity numbers (RIN) > 9 were hybridized to Human Affymetrix Clariom D microarrays. Data were quantile normalized and summarized with Transcriptome Analysis Console (v4.0; Thermo Fisher Scientific) using the SST-RMA algorithm. Annotation of the data was performed using the Affymetrix library for Clariom D Array (version 2, human) at gene level. Data is publicly available under accession numbers GSE294433 and GSE295817 at the Gene Expression Omnibus (GEO).

Differentially expressed genes across shRNAs and cell lines were identified as follows: First, normalized gene expression signal was log_2 transformed. To avoid false discovery artifacts due to the detection of only minimally expressed genes, we excluded all genes with a lower or just minimally higher gene expression signal than that observed for *ERG*, which is known to be virtually not expressed in *EWSR1::FLI1* positive EwS cell lines (27). A log_2FC was calculated for every cell line/construct and normalized to its respective shCtr. Normalized log_2FCs were summarized across cell lines/construct as median log_2FC.

Analysis of publicly available gene expression data and patient survival analysis

Microarray data of 196 EwS tumors (Cohort 1) (GSE63157, GSE34620, GSE12102, GSE17618 and unpublished data (28–31)) for which well-curated clinical annotations were available were downloaded from the GEO. The data were either generated on Affymetrix HG-U133Plus2.0 or on Affymetrix HuEx-1.0-st microarray chips and were normalized separately by RMA using custom brainarray chip description files (CDF, v20) as previously described (32). Batch effects were removed using ComBat (33). Tumor purity was calculated using ESTIMATE (34) and only samples with a tumor purity >60% corresponding to The Cancer Genome Atlas (TCGA) standard (http://cancergenome.nih.gov/cancersselected/biospeccriteria) were kept for further analyses.

Samples were stratified by their quartile intra-tumoral gene expression levels. Significance levels were calculated with a Mantel-Haenszel test (calculated using GraphPad Prism version 9). *P*-values <0.05 were considered as statistically significant.

For analyzing the effect of *EIF4EBP1* expression level on clinical outcome in tumor entities other than EwS we analyzed TCGA RNA-seq unnormalized counts data using TCGAbiolinks R package (21) (STAR - Counts, GRCh38.p0). For downstream analysis we selected all primary tumor samples for which clinical annotation was complete and SNP array and RNA-seq datasets existed. We kept all genes with at least 50% samples showing counts greater than 0. Using DESeq2 (35) for downstream analysis we applied variance stabilizing transformation algorithm on the counts matrix to produce normalized counts on the log2 scale. To investigate the association between *EIF4EBP1* expression and overall patient survival we performed Kaplan Meier analyses using the best percentile approach (23). To determine the optimal threshold based on *EIF4EBP1* expression as a continuous variable (vst-counts), we used maximally selected rank statistics from the maxstat R package (36). Significance levels were calculated using the log-rank test.

Male and female patients were included in the analyses.

Fast gene-set enrichment analysis (fGSEA) and single-sample GSEA (ssGSEA)

fGSEA was performed using the FGSEA R package (v 4.1.3) based on Gene Ontology (GO) biological processes terms from MSigDB (c5.all.v7.5.1 symbols.gmt, c2.all.v7.5.1 symbols.gmt, c2.cgp.v7.5.1 symbols.gmt) and GO terms were filtered for statistical significance (adjusted *P*<0.05) (37, 38).

Using the Affymetrix gene expression dataset comprising 196 EwS patients (Cohort 1), enrichment of gene sets that are among *EIF4EBP1* co-regulated genes were identified by ranking of Pearson´s correlation coefficient of the expression of each gene with *EIF4EBP1* expression and performance of a pre-ranked fGSEA.

For the RNA-seq gene expression dataset comprising 100 primary EwS patients (Cohort 2), enrichment of gene sets that are among *EIF4EBP1* co-regulated genes and among chr8 regulated genes were identified by performing a pre-ranked fGSEA on a) ranked Pearson´s correlation coefficient between the expression of each gene with *EIF4EBP1* expression and on b) ranked mean log_2FC as calculated for each gene between EwS with and without chr8 gain.

Employing datasets from MS protein expression data and microarray gene expression data of A-673, SK-N-MC, and TC-71 EwS cell lines as well as A-673 and TC-71 xenografts with/without 4E-BP1 knockdown, containing differentially expressed proteins upon 4E-BP1 knockdown, all proteins were ranked by their median log_2 FC and a pre-ranked fGSEA was performed.

We applied single-sample Gene Set Enrichment Analysis (ssGSEA) to infer chromosome 8 (chr8) gene enrichment in 196 Ewing sarcoma tumor samples (Cohort 1) (39, 40). The gene set consisted of genes located within the chr8 cytogenetic region (MSigDB v7.4, c1.all.v7.5.symbols.gmt; **Supplementary Table 14**) that were also present within the gene expression matrix (n=439). *MYC* and *RAD21*, as shown in **Supplementary Figure 1c**, are part of respective chr8 signature (**Supplementary Table 18**). The ssGSEA algorithm as implemented in GSVA R package (version 1.50.5) calculated an enrichment score for the chromosome 8 gene set in each individual sample, allowing for a patient-specific assessment of gene set activity. To establish a cut-off between chromosome 8 high and low groups, we employed hierarchical clustering (median linkage, Euclidean distance method) on scaled enrichment scores (range -2,24 to 3,15) dividing samples into 2 cluster: ‘Chr8 high’ (n=117) and ‘Chr8 low’ (n=79). Clustering was visualized using pheatmap R package (version 1.0.12). This approach enabled patient-specific assessment of chr8 gene set activity for comparative analyses.

To refine the chr8 enrichment approach by excluding samples with other recurrent CNVs, the same approach as for chr8 was applied with gene sets reflecting chr1q, chr12, and chr16. Samples were assigned as only chr8 high when none of the other recurrent CNVs were present, and as only chr8 low when no recurrent CNVs were present. This resulted in 117 EwS patients (Cohort 1, chr8 focus)

which were included in this refined analysis. To compare both groups (chr8 high vs. low) regarding overall patient survival we performed a Kaplan Meier analysis. Significance levels were calculated with Mantel-Haenszel test (GraphPad Prism version 9).

Position Related Data Analysis (PREDA)

To validate appropriate clustering of Cohort 1 tumor samples into chr8 high and low gene expression groups we determined differentially expressed genes (DEGs) between both clusters using Limma (41) and Position Related Data Analysis (PREDA) (42) on iDEP (43) platform to map respective DEGs (FDR cut-off < 0.01) onto chromosomes. Using the Limma package (41), also DEGs of the mTOR signaling pathway were depicted.

Cancer Dependency Map (DepMap) analyses

To identify potential therapeutic targets for EwS cells, we leveraged existing curated cancer Dependency Map (DepMap, Broad Institute) data (44–46). For each gene, moderated estimates of the differences between the means of gene dependency effects across EwS (*n*=23) versus all other cell lines (*n*=1077) were plotted against its statistical significance. Q-values were calculated to adjust for multiple testing.

STRING analysis

The STRING (v.12.0) protein interactions network database was used to obtain determine direct (physical) and indirect (functional) associations (47) between the following proteins: EIF4EBP1, CDK4, CDK6, CCND1, CDC25B, PRMT5, MCM2, RBL1, RNF2, and USP14. The online tool was used in default setting.

Histology

HE-staining of EwS xenografts was performed according to routine protocols. Mitoses of EwS xenografts were quantified in HE-stained slides by two blinded observers in 5 high-power fields per sample. Mitoses per sample were determined by mean of total 10 counted high-power fields across both observers.

Statistics and software

Statistical data analysis was performed using GraphPad PRISM 9 (GraphPad Software Inc., CA, USA) or with R (version 4.2.0) on the raw data. If not otherwise specified in the Figure legends, comparison of two groups in functional *in vitro* experiments was carried out using a two-tailed Mann-Whitney test. If not otherwise specified in the Figure legends, data are presented as dot plots with horizontal bars representing means, and whiskers representing the standard error of the mean (SEM). Sample size for all *in vitro* experiments was chosen empirically with at least 3 biological replicates. In Kaplan-Meier overall survival analyses, curves were calculated from all individual survival times of patients. Curves were compared by Mantel-Haenszel test to detect significant differences between the groups. For batch analyses of patient survival, the in-house custom code software GenEx was used, using the Mantel-Haenszel test for *P*-value calculation. Pearson´s correlation coefficients were calculated using Microsoft Excel or GraphPad PRISM 9 (GraphPad Software Inc., CA, USA). For *in vivo* experiments, sample size was predetermined using power calculations with $\beta = 0.8$ and $\alpha=0.05$ based on preliminary data and in compliance with the 3R system (replacement, reduction, refinement). Kaplan-Meier analyses of event-free survival (*in vivo* experiments) were carried out using GraphPad PRISM 9 (GraphPad Software Inc.). The definition of the type of events is given in the corresponding Figure legends but generally corresponds to humane experimental endpoints as defined above.

REFERENCES

1. Livak KJ, Schmittgen TD. Analysis of relative gene expression data using real-time quantitative PCR and the 2(-Delta Delta C(T)) Method. *Methods San Diego Calif.* 2001;25(4):402–408.

2. Wiederschain D, et al. Single-vector inducible lentiviral RNAi system for oncology target validation. *Cell Cycle Georget Tex*. 2009;8(3):498–504.

3. Marchetto A, Romero-Pérez L. Western Blot Analysis in Ewing Sarcoma. *Methods Mol Biol Clifton NJ*. 2021;2226:15–25.

4. Funk CM, Musa J. Proliferation Assessment by Trypan Blue Exclusion in Ewing Sarcoma. *Methods Mol Biol Clifton NJ*. 2021;2226:151–158.

5. Schindelin J, et al. Fiji: an open-source platform for biological-image analysis. *Nat Methods*. 2012;9(7):676–682.

6. Schneider CA, Rasband WS, Eliceiri KW. NIH Image to ImageJ: 25 years of image analysis. *Nat Methods*. 2012;9(7):671–675.

7. ORFeome Collaboration. The ORFeome Collaboration: a genome-scale human ORF-clone resource. *Nat Methods*. 2016;13(3):191–192.

8. Peterziel H, et al. Drug sensitivity profiling of 3D tumor tissue cultures in the pediatric precision oncology program INFORM. *NPJ Precis Oncol*. 2022;6(1):94.

9. Berker Y, et al. Patient-by-Patient Deep Transfer Learning for Drug-Response Profiling Using Confocal Fluorescence Microscopy of Pediatric Patient-Derived Tumor-Cell Spheroids. *IEEE Trans Med Imaging*. 2022;41(12):3981–3999.

10. ElHarouni D, et al. iTReX: Interactive exploration of mono- and combination therapy dose response profiling data. *Pharmacol Res*. 2022;175:105996.

11. van Tilburg CM, et al. The Pediatric Precision Oncology INFORM Registry: Clinical Outcome and Benefit for Patients with Very High-Evidence Targets. *Cancer Discov*. 2021;11(11):2764–2779.

12. Müller T, et al. Automated sample preparation with SP3 for low-input clinical proteomics. *Mol Syst Biol*. 2020;16(1):e9111.

13. Müller T, et al. Automated Sample Preparation for Mass Spectrometry-Based Clinical Proteomics. *Methods Mol Biol Clifton NJ*. 2023;2718:181–211.

14. Cidre-Aranaz F, Ohmura S. Tumor Growth Analysis of Ewing Sarcoma Cell Lines Using Subcutaneous Xenografts in Mice. *Methods Mol Biol Clifton NJ*. 2021;2226:191–199.

15. Kendsersky NM, et al. The B7-H3-Targeting Antibody-Drug Conjugate m276-SL-PBD Is Potently Effective Against Pediatric Cancer Preclinical Solid Tumor Models. *Clin Cancer Res Off J Am Assoc Cancer Res*. 2021;27(10):2938–2946.

16. Hingorani P, et al. Trastuzumab Deruxtecan, Antibody-Drug Conjugate Targeting HER2, Is Effective in Pediatric Malignancies: A Report by the Pediatric Preclinical Testing Consortium. *Mol Cancer Ther*. 2022;21(8):1318–1325.

17. Ghilu S, et al. Approaches to identifying drug resistance mechanisms to clinically relevant treatments in childhood rhabdomyosarcoma. *Cancer Drug Resist*. 2022;5(1):80–89.

18. Murphy B, et al. Evaluation of Alternative In Vivo Drug Screening Methodology: A Single Mouse Analysis. *Cancer Res*. 2016;76(19):5798–5809.

19. Makvandi M, et al. Pre-clinical investigation of astatine-211-parthanatine for high-risk neuroblastoma. *Commun Biol*. 2022;5(1):1–9.

20. Koelsche C, et al. Sarcoma classification by DNA methylation profiling. *Nat Commun*. 2021;12(1):498.

21. Colaprico A, et al. TCGAbiolinks: an R/Bioconductor package for integrative analysis of TCGA data. *Nucleic Acids Res*. 2016;44(8):e71.

22. Franch-Expósito S, et al. CNApp, a tool for the quantification of copy number alterations and integrative analysis revealing clinical implications. *eLife*;9:e50267.

23. Therneau TM, Grambsch PM. *Modeling Survival Data: Extending the Cox Model*. Springer Science & Business Media; 2013.

24. Therneau TM, et al. survival: Survival Analysis. 2023. https://cran.r-project.org/web/packages/survival/index.html. Accessed September 10, 2023.

25. Stichel D, et al. Routine RNA sequencing of formalin-fixed paraffin-embedded specimens in neuropathology diagnostics identifies diagnostically and therapeutically relevant gene fusions. *Acta Neuropathol (Berl)*. 2019;138(5):827–835.

26. McPherson A, et al. deFuse: an algorithm for gene fusion discovery in tumor RNA-Seq data. *PLoS Comput Biol*. 2011;7(5):e1001138.

27. Crompton BD, et al. The genomic landscape of pediatric Ewing sarcoma. *Cancer Discov*. 2014;4(11):1326–1341.

28. Savola S, et al. High Expression of Complement Component 5 (C5) at Tumor Site Associates with Superior Survival in Ewing's Sarcoma Family of Tumour Patients. *ISRN Oncol*. 2011;2011:168712.

29. Postel-Vinay S, et al. Common variants near TARDBP and EGR2 are associated with susceptibility to Ewing sarcoma. *Nat Genet*. 2012;44(3):323–327.

30. Scotlandi K, et al. Overcoming resistance to conventional drugs in Ewing sarcoma and identification of molecular predictors of outcome. *J Clin Oncol Off J Am Soc Clin Oncol*. 2009;27(13):2209–2216.

31. Volchenboum SL, et al. Gene Expression Profiling of Ewing Sarcoma Tumors Reveals the Prognostic Importance of Tumor-Stromal Interactions: A Report from the Children's Oncology Group. *J Pathol Clin Res*. 2015;1(2):83–94.

32. Sannino G, et al. Gene expression and immunohistochemical analyses identify SOX2 as major risk factor for overall survival and relapse in Ewing sarcoma patients. *EBioMedicine*. 2019;47:156–162.

33. Johnson WE, Li C, Rabinovic A. Adjusting batch effects in microarray expression data using empirical Bayes methods. *Biostat Oxf Engl*. 2007;8(1):118–127.

34. Yoshihara K, et al. Inferring tumour purity and stromal and immune cell admixture from expression data. *Nat Commun*. 2013;4:2612.

35. Love MI, Huber W, Anders S. Moderated estimation of fold change and dispersion for RNA-seq data with DESeq2. *Genome Biol*. 2014;15(12):550.

36. Hothorn T. maxstat: Maximally Selected Rank Statistics. 2017. https://cran.r-project.org/web/packages/maxstat/index.html. Accessed September 10, 2023.

37. Korotkevich G, et al. Fast gene set enrichment analysis [preprint]. 2021;060012.

38. Subramanian A, et al. Gene set enrichment analysis: a knowledge-based approach for interpreting genome-wide expression profiles. *Proc Natl Acad Sci U S A*. 2005;102(43):15545–15550.

39. Hänzelmann S, Castelo R, Guinney J. GSVA: gene set variation analysis for microarray and RNA-seq data. *BMC Bioinformatics*. 2013;14:7.

40. Barbie DA, et al. Systematic RNA interference reveals that oncogenic KRAS-driven cancers require TBK1. *Nature*. 2009;462(7269):108–112.

41. Ritchie ME, et al. limma powers differential expression analyses for RNA-sequencing and microarray studies. *Nucleic Acids Res*. 2015;43(7):e47.

42. Ferrari F, et al. PREDA: an R-package to identify regional variations in genomic data. *Bioinforma Oxf Engl*. 2011;27(17):2446–2447.

43. Ge SX, Son EW, Yao R. iDEP: an integrated web application for differential expression and pathway analysis of RNA-Seq data. *BMC Bioinformatics*. 2018;19(1):534.

44. Dempster JM, et al. Chronos: a cell population dynamics model of CRISPR experiments that improves inference of gene fitness effects. *Genome Biol*. 2021;22(1):343.

45. Dempster JM, et al. *Extracting Biological Insights from the Project Achilles Genome-Scale CRISPR Screens in Cancer Cell Lines*. Cancer Biology; 2019.

46. Meyers RM, et al. Computational correction of copy-number effect improves specificity of CRISPR-Cas9 essentiality screens in cancer cells. *Nat Genet*. 2017;49(12):1779–1784.

47. Szklarczyk D, et al. The STRING database in 2023: protein-protein association networks and functional enrichment analyses for any sequenced genome of interest. *Nucleic Acids Res*. 2023;51(D1):D638–D646.

Supplementary Figure 1 Funk and Ehlers *et al.* 2025

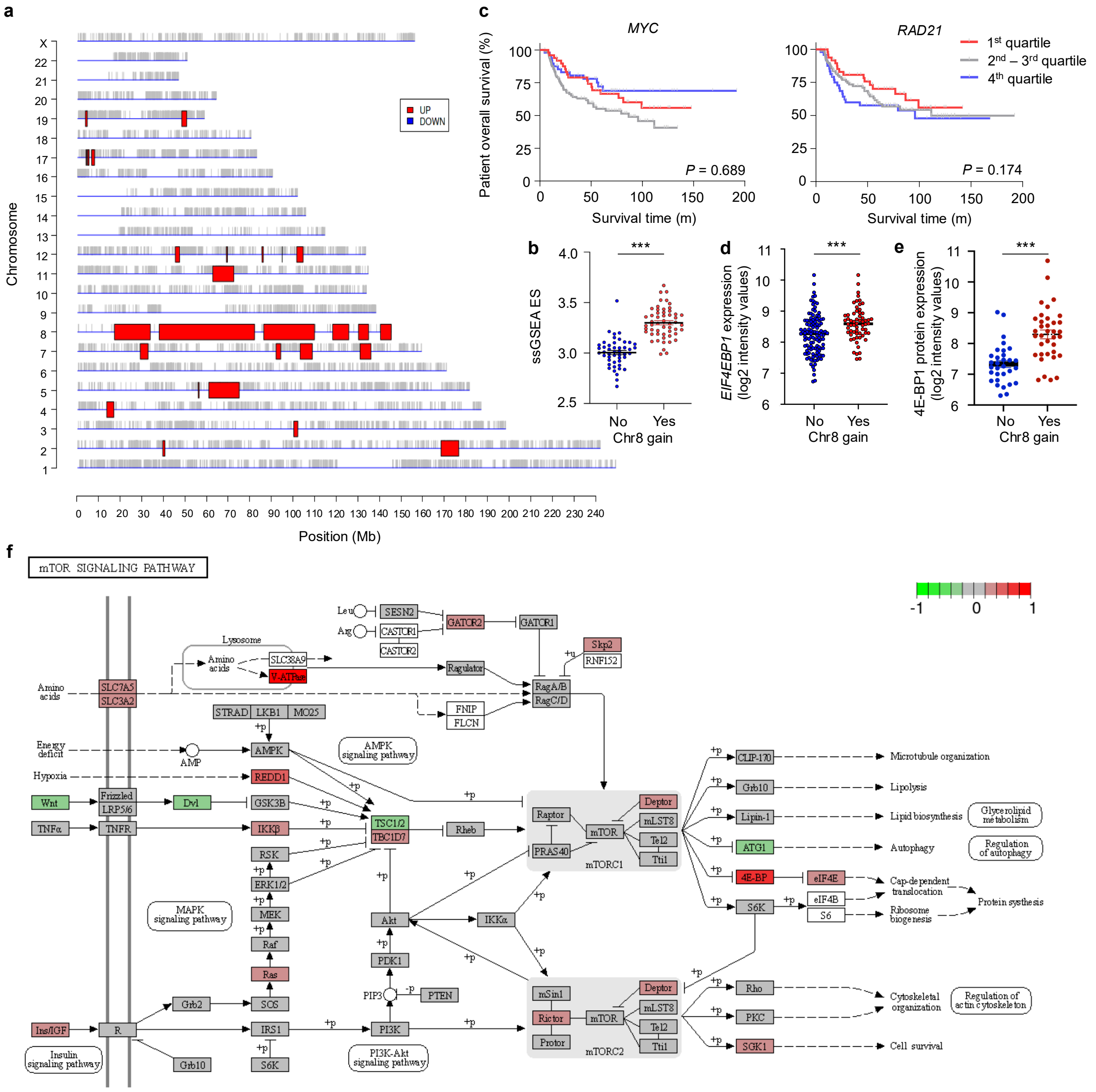

Supplementary Figure 1: Model validation, chromosomal location, and mTOR pathway representation of genes differentially upregulated in the chr8 high gene expression signature group in EwS.

a Differentially expressed genes (DEGs) between the chr8 high and low gene enrichment clusters mapped onto chromosome positions using Position Related Data Analysis (PREDA).

b ssGSEA enrichment scores for chr8 gene expression enrichment as measured by RNA-seq in 100 primary EwS (Cohort 2) depending on the presence of factual chr8 gain as determined by methylation array.

c Kaplan-Meier overall survival analysis of 196 EwS patients (Cohort 1) stratified by quartile *MYC* or RAD21 expression. *P*-values determined by Mantel-Haenszel test.

d *EIF4EBP1* expression as measured by microarray profiling in 117 EwS patients (Cohort 1, Chr8 focus) stratified into either a high or low chr8 signature enrichment group as shown in (Figure 1e) but excluding samples with other inferred recurrent CNVs. *P*-values determined by two-tailed Mann-Whitney test, horizontal bars represent means and whiskers represent the SEM.

e 4E-BP1 protein expression as measured by mass spectrometry in a subset of Cohort 2 for which 4E-BP1 protein expression data were available. 4E-BP1 expression is shown depending on the evidence of chr8 gain in methylation array data. *P*-values determined by two-tailed Mann-Whitney test, horizontal bars represent means and whiskers represent the SEM.

f DEGs between the chr8 high and low gene expression cluster in Cohort 1 within the mTOR signaling pathway.

***$P < 0.001$, *P*-values determined via two-tailed Mann-Whitney test.

Supplementary Figure 2 Funk and Ehlers *et al.* 2025

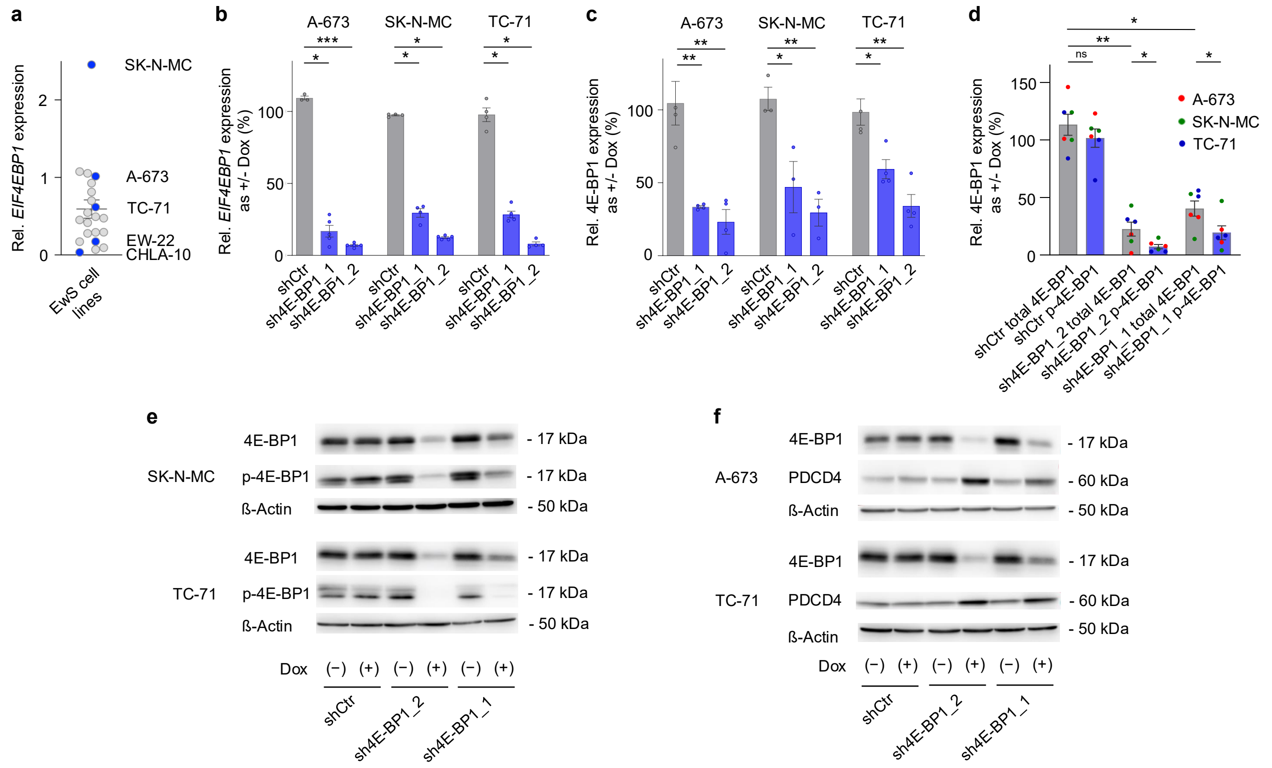

Supplementary Figure 2: 4E-BP1 drives a proliferation-associated proteomic network.

a Relative *EIF4EBP1* expression in 21 wildtype EwS cell lines as determined by qRT-PCR. *EIF4EBP1* expression of each cell line is normalized to that of A-673.

b Relative *EIF4EBP1* expression as assessed by qRT-PCR in A-673, SK-N-MC, and TC-71 cells containing either Dox-inducible specific shRNA constructs directed against *EIF4EBP1* (sh4E-BP1_1 or sh4E-BP1_2) or a non-targeting shControl (shCtr). Cells were grown either with or without Dox for 96 h. Horizontal bars represent means, and whiskers represent the SEM, n≥3 biologically independent experiments. *P*-values determined via two-tailed Mann-Whitney test and adjusted for multiple comparisons with the Benjamini-Hochberg method.

c Relative 4E-BP1 expression as assessed by quantified western blotting in A-673, SK-N-MC, and TC-71 cells containing either Dox-inducible specific shRNA constructs directed against *EIF4EBP1* (sh4E-BP1_1 or sh4E-BP1_2) or a non-targeting shControl (shCtr). Cells were grown either with or without Dox for 96 h. *P*-values determined via unpaired t-test and adjusted for multiple comparisons with the Benjamini-Hochberg method.

d Relative total and phospho (Ser65) 4E-BP1 expression as assessed by quantified western blotting in A-673, SK-N-MC, and TC-71 cells containing either Dox-inducible specific shRNA constructs directed against *EIF4EBP1* (sh4E-BP1_1 or sh4E-BP1_2) or a non-targeting shControl (shCtr). Cells were grown either with or without Dox for 96 h. *P*-values determined via one-tailed Mann-Whitney test and adjusted for multiple comparisons with the Benjamini-Hochberg method.

e Representative western blots in SK-N-MC and TC-71 cells of experiments described in (d). ß-Actin served as a loading control.

f Representative western blots showing total 4E-BP1 and PDCD4 expression levels in A-673 and TC-71 cells containing either Dox-inducible specific shRNA constructs directed against *EIF4EBP1* (sh4E-BP1_1 or sh4E-BP1_2) or a non-targeting shControl (shCtr). Cells were grown either with or without Dox for 96 h. ß-Actin served as a loading control.

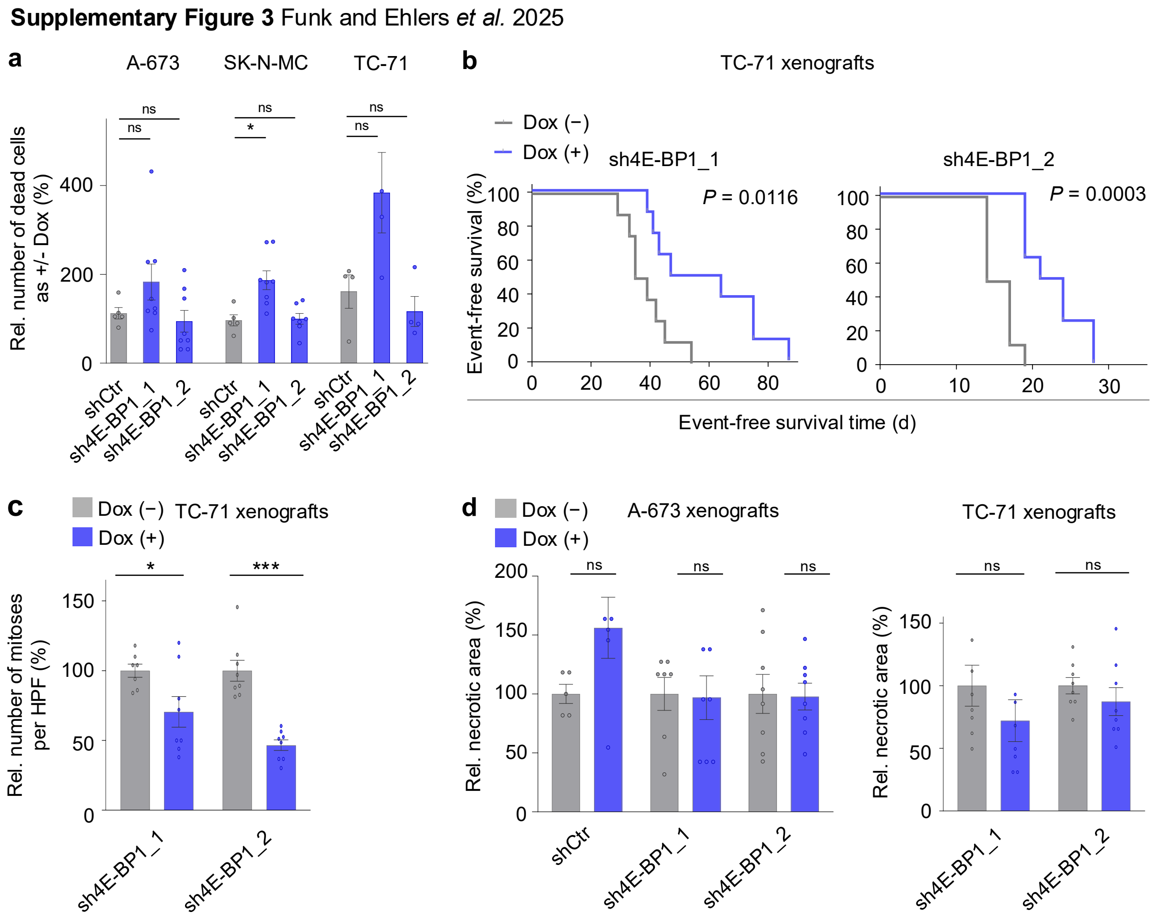

Supplementary Figure 3: RNAi-mediated knockdown of 4E-BP1 inhibits EwS growth.

a Relative number of dead cells as assessed by Trypan blue exclusion in A-673, SK-N-MC, and TC-71 cells containing either Dox-inducible specific shRNA constructs directed against *EIF4EBP1* (sh4E-BP1_1 or sh4E-BP1_2) or a non-targeting shControl (shCtr). Cells were grown either with or without Dox for 96 h. Horizontal bars represent means, and whiskers represent the SEM, n≥4 biologically independent experiments. *P*-values determined via two-tailed Mann-Whitney test and adjusted for multiple comparisons with the Benjamini-Hochberg method.

b Kaplan-Meier analysis of event-free survival of NSG mice xenografted with TC-71 cells containing Dox-inducible specific shRNA constructs directed against *EIF4EBP1* (sh4E-BP1_1 or sh4E-BP1_2). Once tumors were palpable, mice were randomized and treated with either vehicle (–) or Dox (+), n =8 animals per condition. An 'event' was recorded when tumors reached a size maximum of 15 mm in one dimension. *P*-values determined via Mantel-Haenszel test.

c Quantification of mitoses in HE-stained slides of xenografts described in (b). Five high-power fields (HPF) were counted per sample. Horizontal bars represent means, and whiskers represent the SEM, n ≥ 7 samples per condition.

d Quantification of necrotic area on HE-stained slides of A-673 and TC-71 xenografts described in (Fig. 3d, Suppl. Fig. 3b). Five high-power fields (HPF) were analyzed per sample. Horizontal bars represent means, and whiskers represent the SEM, n≥5 samples per condition.

Supplementary Figure 4 Funk and Ehlers *et al.* 2025

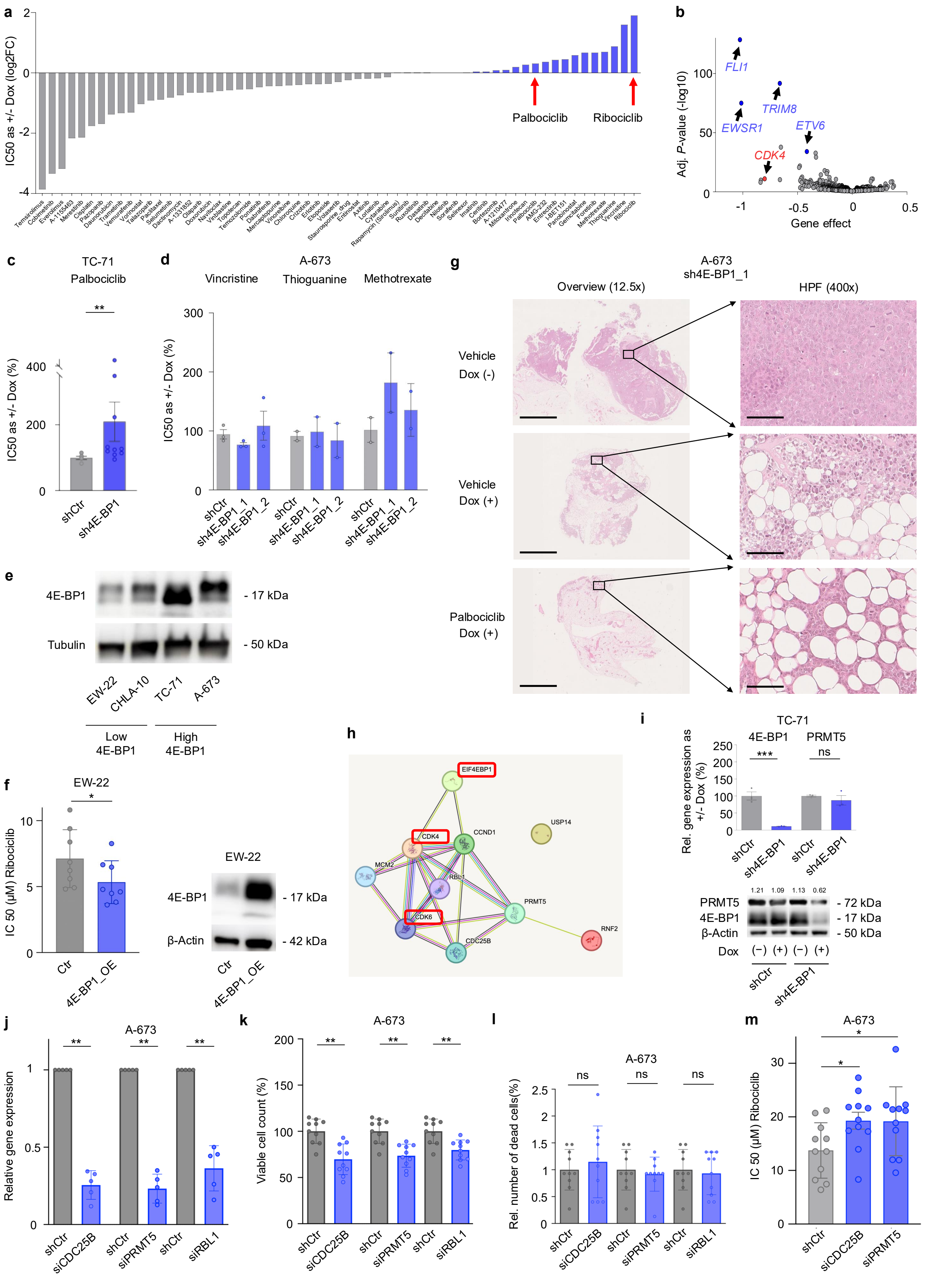

Supplementary Figure 4: High 4E-BP1 expression sensitizes for targeted CDK4/6 inhibitor treatment with Palbociclib and Ribociclib.

a IC50 analysis of 3D culture drug screening data of A-673 EwS cells containing a Dox-inducible shRNA directed against 4E-BP1 and treated with/without Dox and respective indicated inhibitors in serially increasing concentrations.
b Volcano plot showing gene dependency effects of indicated genes in EwS cell lines as compared to all non-EwS cell lines with respective individual statistical significance values (-log10 adj. *P*-value).
c IC50 analysis of CDK4/6 inhibitor Palbociclib in TC-71 cells containing either DOX-inducible specific shRNAs directed against 4E-BP1, or a non-targeting a non-targeting shControl (shCtr) as measured by resazurin colorimetry. Cells were treated with/without Dox as well as with serial dilutions of the inhibitors. Horizontal bars represent means, and whiskers represent the SEM, $n \geq 5$ biologically independent experiments.
d IC50 analysis of Vincristine, Thioguanine, and Methotrexate in A-673 cells containing either DOX-inducible specific shRNAs directed against 4E-BP1 (sh4E-BP1_1, sh4E-BP1_2) or a non-targeting a non-targeting shControl (shCtr) as measured by resazurin colorimetry. Cells were treated with/without Dox as well as with serial dilutions of respective drugs. Horizontal bars represent means, and whiskers represent the SEM, $n \geq 2$ biologically independent experiments.
e Representative western blots in EW-22, CHLA-10 (low 4E-BP1 expressing) and TC-71 and A-673 (high 4E-BP1 expressing) EwS cells. ß-Actin served as a loading control.
f IC50 analysis of Ribociclib in EW-22 cells containing either a 4E-BP1 overexpression construct (4E-BP1_OE) or a negative control (Ctr) construct as measured by resazurin colorimetry (left). Cells were treated with a serial dilution of Ribociclib. Horizontal bars represent means, and whiskers represent the SEM, n=8 biologically independent experiments. Representative western blots in EW-22 cells containing either a 4E-BP1 overexpression construct (4E-BP1_OE) or a negative control (Ctr) construct (right). ß-Actin served as a loading control.
g Representative HE stained micrographs of A673/sh4E-BP1 xenografts (Dox (–) / Dox (+)) treated with either vehicle or Palbociclib as described in (Figure 4c) (shown as an overview with 12.5× magnification and as a high-power field (HPF) in 400× magnification). Scale bar is 2.5 mm (12.5×) and 100 µm (400×).
h STRING interaction analysis of the following proteins: EIF4EBP1, CDK4, CDK6, CCND1, CDC25B, PRMT5, MCM2, RBL1, RNF2, and USP14. Color codes of lines and nodes indicate form of interaction as defined by the STRING database. EIF4EBP1 and CDK4/6 are highlighted in red rectangles.
i Upper panel: Relative *EIF4EBP1* and *PRMT5* expression as assessed by qRT-PCR in TC-71 cells containing either Dox-inducible specific shRNA constructs directed against *EIF4EBP1* (sh4E-BP1) or a non-targeting shControl (shCtr). Cells were grown either with or without Dox for 96 h. Horizontal bars represent means, and whiskers represent the SEM, n=3 biologically independent experiments. Lower panel: Representative corresponding western blots of the same experiments using antibodies against 4E-BP1 and PRMT5. Numbers indicate the densitometry ratios of PRMT5 normalized to ß-Actin. ß-Actin served as a loading control.
j Relative expression of *CDC25B*, *PRMT5*, and *RBL1* as assessed by qRT-PCR in A-673 cells treated either with siPools directed against respective genes or a negative control siPool. Horizontal bars represent means, and whiskers represent the SEM, n=5 biologically independent experiments.
k Relative viable cell count of A-673 cells treated either with siPools directed against *CDC25B*, *PRMT5*, or *RBL1*, or a negative control siPools as measured by Trypan blue exclusion. Cells were assayed 120 h after knockdown induction. Horizontal bars represent means, and whiskers represent the SEM, n=10 biologically independent experiments.
l Relative number of dead cells in experiments detailed in (**i**) as measured by Trypan blue exclusion. Horizontal bars represent means, and whiskers represent the SEM, n=10 biologically independent experiments. *P*-values determined via two-tailed Mann-Whitney test and adjusted for multiple comparisons with the Benjamini-Hochberg method.
m IC50 analysis of Ribociclib in A-673 cells treated either with siPools directed against *CDC25B* or *PRMT5*, or a negative control siPool as measured by resazurin colorimetry. Cells were additionally treated with a serial dilution of Ribociclib. Horizontal bars represent means, and whiskers represent the SEM, n=11 biologically independent experiments.
***$P < 0.001$, **$P < 0.01$, *$P < 0.05$, ns = not significant; *P*-values determined via two-tailed Mann-Whitney test if not otherwise specified.
