# Supplementary material for: High 4E-BP1 expression associates with chromosome 8 gain and CDK4/6 sensitivity in Ewing sarcoma
Source: J Clin Invest. 2025 Oct 16;135(24):e187627. doi: 10.1172/JCI187627 (PMC12700544; doi:10.1172/JCI187627)

Funk and Ehlers *et al.* 2025
Unedited blots, Figure 2c

A-673, total 4E-BP1

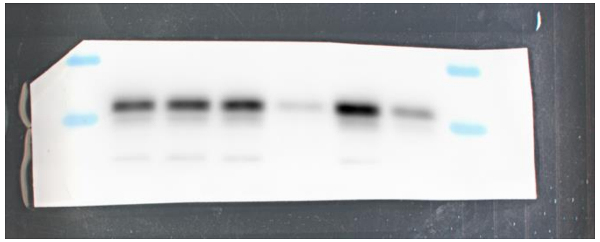

SK-N-MC, total 4E-BP1

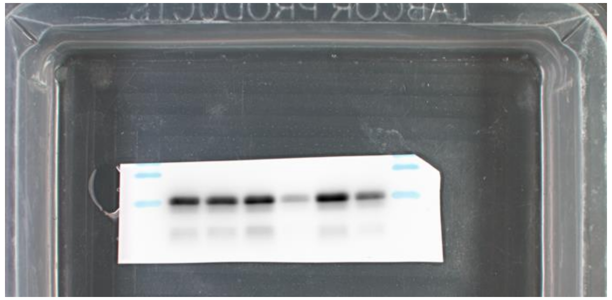

TC-71, total 4E-BP1

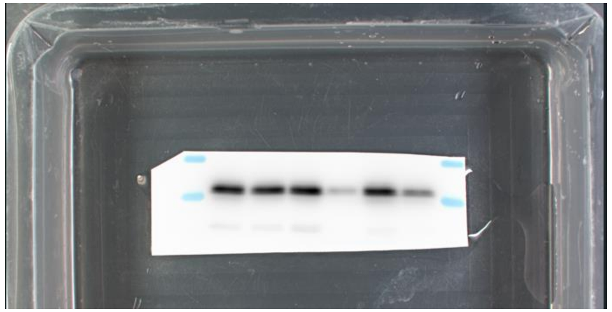

A-673, ß-Actin

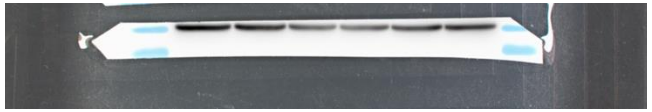

SK-N-MC, ß-Actin

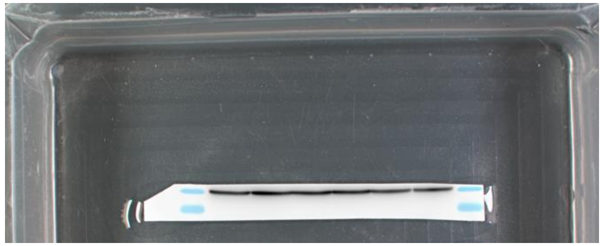

TC-71, ß-Actin

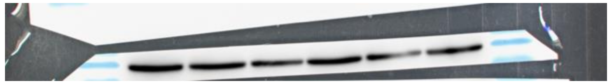

Funk and Ehlers *et al.* 2025
Unedited blots, Supplementary Figure 2e

SK-N-MC, total 4E-BP1

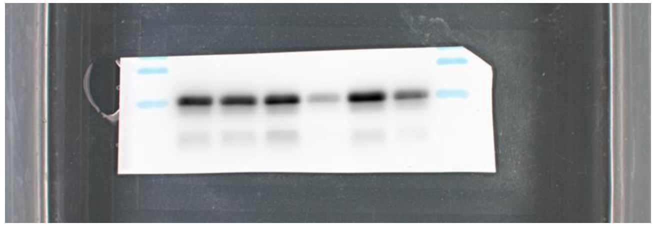

SK-N-MC, p-4E-BP1

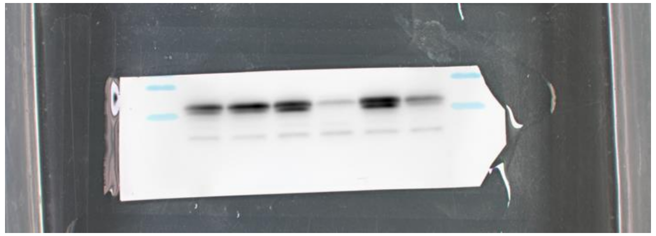

SK-N-MC, ß-Actin

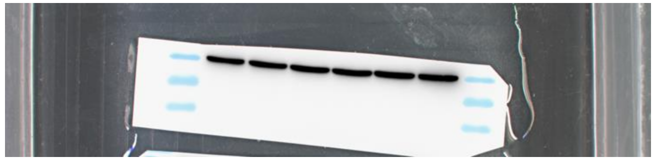

TC-71, total 4E-BP1

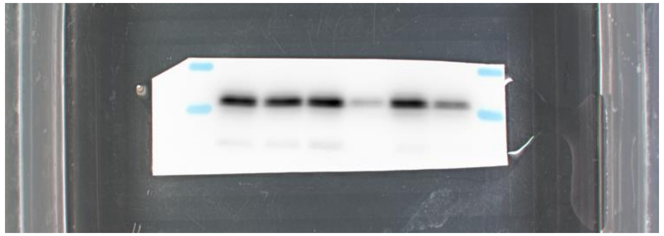

TC-71, p-4E-BP1

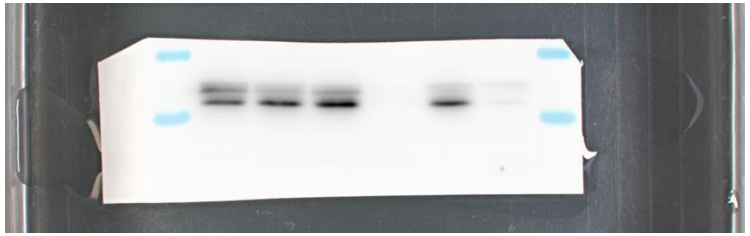

TC-71, ß-Actin

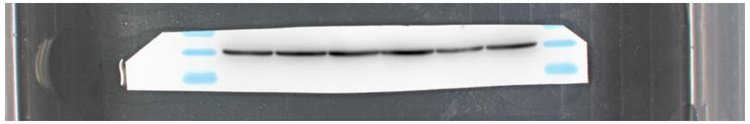

Funk and Ehlers *et al.* 2025
Unedited blots, Supplementary Figure 2f

A-673, total 4E-BP1

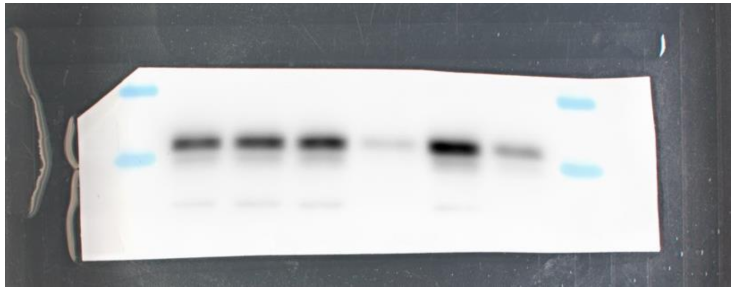

TC-71, total 4E-BP1

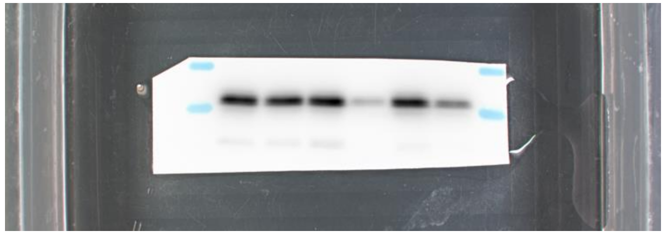

A-673, PDCD4

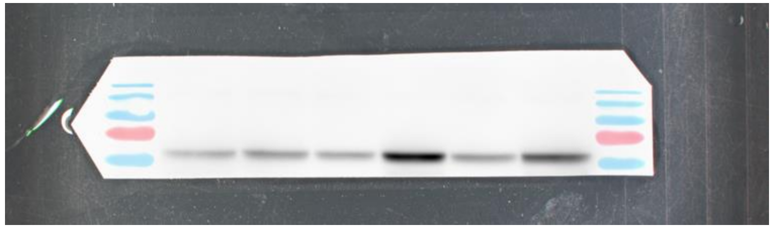

TC-71, PDCD4

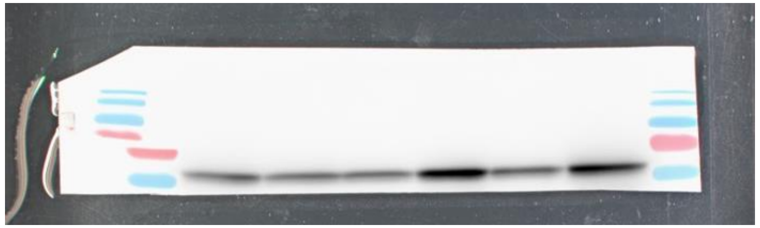

A-673, ß-Actin

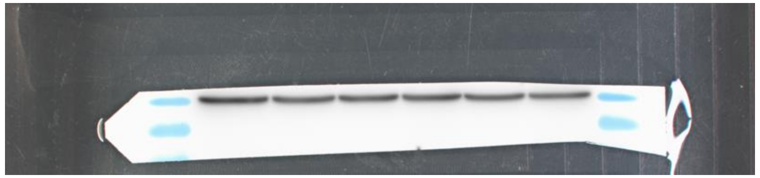

TC-71, ß-Actin

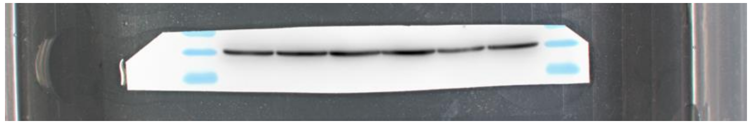

Funk and Ehlers *et al.* 2025
Unedited blots, Supplementary Figure 4e

4E-BP1

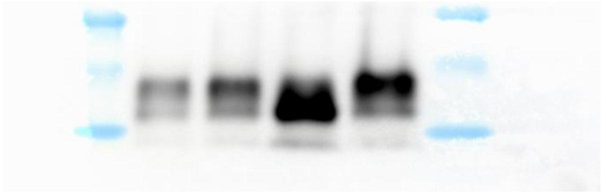

EW-22 CHLA-10 TC-71 A-673

Tubulin

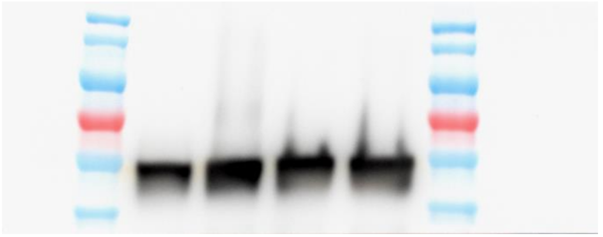

EW-22 CHLA-10 TC-71 A-673

Funk and Ehlers *et al.* 2025
Unedited blots, Supplementary Figure 4g

EW-22, 4E-BP1

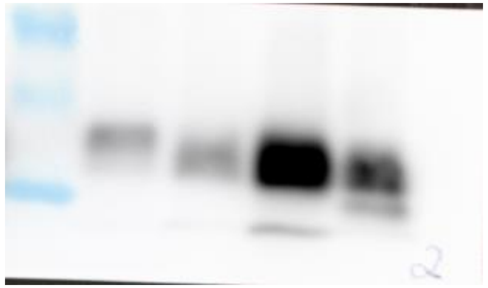

EW22, ß-Actin

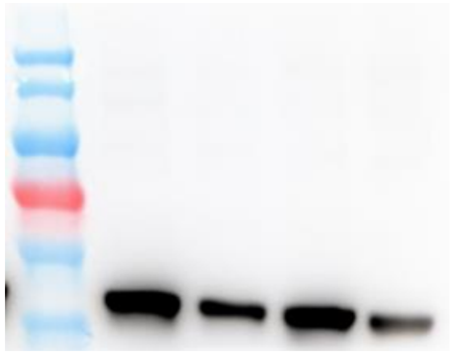

Funk and Ehlers *et al.* 2025
Unedited blots, Supplementary Figure 4i

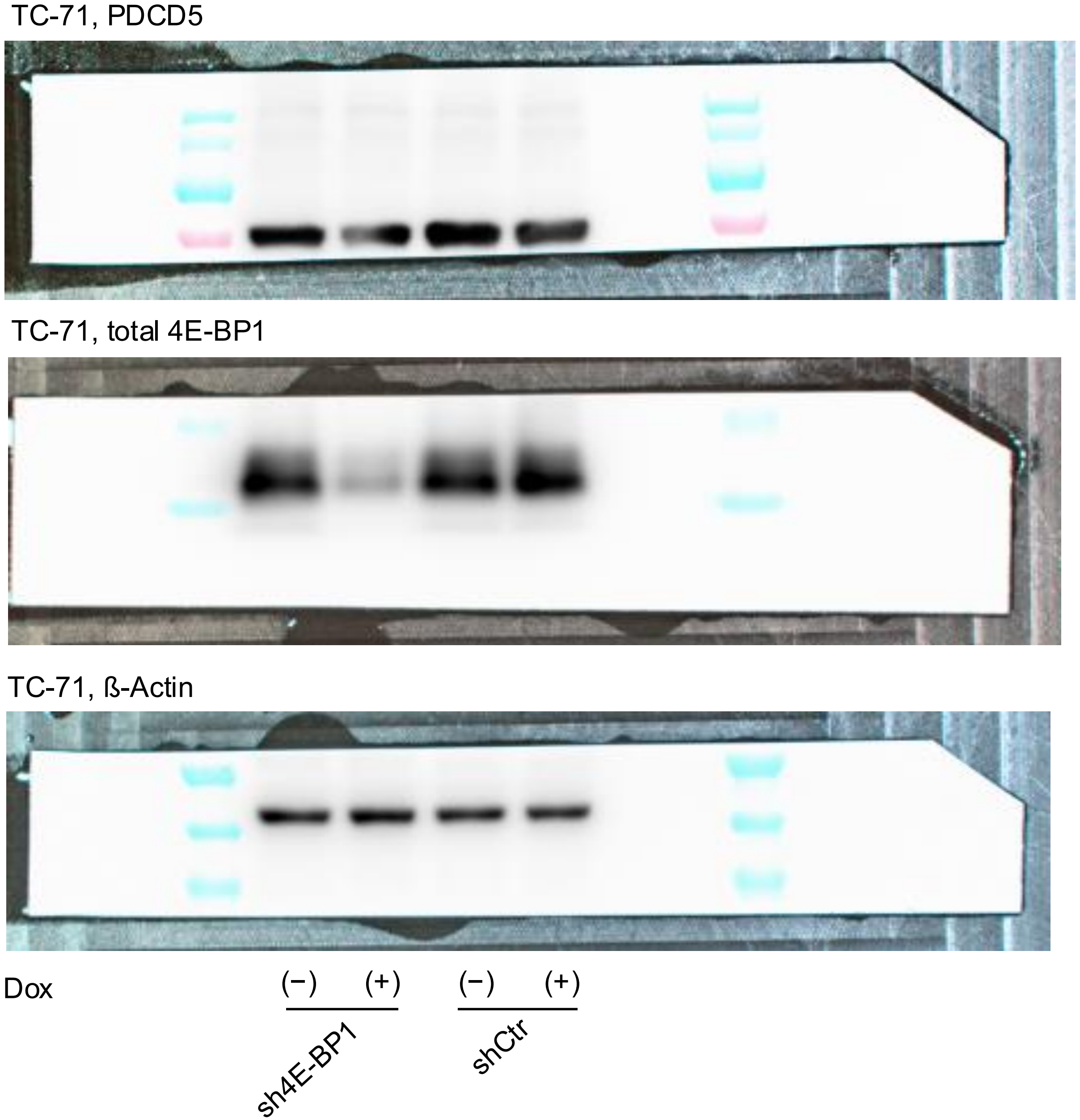

Supplement: Unedited blot and gel images [file jci-135-187627-s287.pdf]
